# Identification of novel molecular subtypes and a signature to predict prognosis and therapeutic response based on cuproptosis-related genes in prostate cancer

**DOI:** 10.3389/fonc.2023.1162653

**Published:** 2023-05-02

**Authors:** Jili Zhang, Shaoqin Jiang, Di Gu, Wenhui Zhang, Xianqi Shen, Min Qu, Chenghua Yang, Yan Wang, Xu Gao

**Affiliations:** ^1^ Department of Urology, Changhai Hospital, Second Military Medical University, Shanghai, China; ^2^ Department of Urology, Fujian Union Hospital, Fujian Medical University, Fuzhou, Fujian, China

**Keywords:** prostate cancer, cuproptosis, unsupervised clustering, tumor microenvironment, signature

## Abstract

**Background:**

Prostate cancer (PCa) is the most common malignant tumor of the male urinary system. Cuproptosis, as a novel regulated cell death, remains unclear in PCa. This study aimed to investigate the role of cuproptosis-related genes (CRGs) in molecular stratification, prognostic prediction, and clinical decision-making in PCa.

**Methods:**

Cuproptosis-related molecular subtypes were identified by consensus clustering analysis. A prognostic signature was constructed with LASSO cox regression analyses with 10-fold cross-validation. It was further validated in the internal validation cohort and eight external validation cohorts. The tumor microenvironment between the two risk groups was compared using the ssGSEA and ESTIMATE algorithms. Finally, qRT-PCR was used to explore the expression and regulation of these model genes at the cellular level. Furthermore, 4D Label-Free LC-MS/MS and RNAseq were used to investigate the changes in CRGs at protein and RNA levels after the knockdown of the key model gene B4GALNT4.

**Results:**

Two cuproptosis-related molecular subtypes with significant differences in prognoses, clinical features, and the immune microenvironment were identified. Immunosuppressive microenvironments were associated with poor prognosis. A prognostic signature comprised of five genes (B4GALNT4, FAM83D, COL1A, CHRM3, and MYBPC1) was constructed. The performance and generalizability of the signature were validated in eight completely independent datasets from multiple centers. Patients in the high-risk group had a poorer prognosis, more immune cell infiltration, more active immune-related functions, higher expression of human leukocyte antigen and immune checkpoint molecules, and higher immune scores. In addition, anti-PDL-1 immunotherapy prediction, somatic mutation, chemotherapy response prediction, and potential drug prediction were also analyzed based on the risk signature. The validation of five model genes' expression and regulation in qPCR was consistent with the results of bioinformatics analysis. Transcriptomics and proteomics analyses revealed that the key model gene B4GALNT4 might regulate CRGs through protein modification after transcription.

**Conclusion:**

The cuproptosis-related molecular subtypes and the prognostic signature identified in this study could be used to predict the prognosis and contribute to the clinical decision-making of PCa. Furthermore, we identified a potential cuproptosis-related oncogene B4GALNT4 in PCa, which could be used as a target to treat PCa in combination with cuproptosis.

## 1 Introduction

Globally, prostate cancer (PCa) accounts for about 1.4 million new cases and 375,000 deaths yearly, making it the second most common cancer and the most common malignant tumor of the male urinary system (1). For patients with localized cancer, radical prostatectomy or radical radiotherapy is the standard treatment (2). Unfortunately, about 20-30% of patients will develop biochemical recurrence after radical treatment, followed by clinical recurrences and metastases (3–5). For advanced PCa, androgen deprivation therapy (ADT) remains the preferred treatment, inhibiting PCa growth by reducing circulating testosterone and inhibiting androgen receptor function (5, 6). However, due to the resistance to ADT, almost all patients progress to castration-resistant PCa (CRPC) after 1 to 2 years of ADT treatment (7). So far, there is no effective treatment for CRPC, and patients usually die within 2-4 years (8, 9). Therefore, it is urgent to explore further the underlying progression mechanisms and new therapeutic targets for advanced PCa.

Although prostate specific antigen level, Gleason score, AJCC TNM staging, and other clinicopathological features have provided important references for monitoring the disease progression and predicting the prognosis of PCa patients (10, 11), the predictive value of these routine features is often limited for patients with an unclear clinical diagnosis or in intermediate grades or stages (12). Furthermore, emerging treatments such as neoadjuvant therapy, chemotherapy, targeted therapy, radionuclide therapy, and immunotherapy have achieved some efficacy in advanced PCa. However, the survival gains from these treatments are unclear for some patients, and these treatments may even lead to severe complications (9). Therefore, due to the heterogeneity of PCa, a reliable prediction tool is required to accurately evaluate the prognosis of patients, which can help clinicians choose the best treatment and determine whether to proceed with more aggressive treatment.

Regulated cell death (RCD), which also refers to programmed cell death (PCD), is a form of cell death that can be regulated by various biological macromolecules (13). In recent years, an increasing number of RCD forms, including apoptosis, necroptosis, autophagy, ferroptosis, and pyroptosis, have been proven to be involved in various pathological and physiological processes, including tumorigenesis (14). Apoptosis, the earliest and most well-studied form of RCD, is the treatment target of almost all tumors (15, 16). However, resistance to apoptosis may be the main reason for the failure of these therapeutic strategies (17). Therefore, it is necessary to discover a new form of RCD and to study its role in tumorigenesis in depth.

Copper, a trace metal, plays a vital role in many biological processes, and maintaining its homeostasis in living organisms is necessary for life (18, 19). On the one hand, copper deficiency in cells can destroy the function of copper-binding enzymes; on the other hand, copper accumulation leads to cell death (20). It has been shown that dysregulation of copper homeostasis contributes to cancer growth, angiogenesis, and metastasis (21). A recent study clarified that excessive copper binds directly to lipoylated components of the tricarboxylic acid (TCA) cycle (22), leading to aggregation of the lipoylated protein and then the loss of iron-sulfur cluster protein, which ultimately kills cells after proteotoxic stress (23). Unlike any other, this novel form of RCD was called “cuproptosis”. Recent studies have shown that cuproptosis is closely associated with the tumor microenvironment (TME) and prognosis of various tumors, including bladder cancer, hepatocellular carcinoma, breast cancer and melanoma (24–27). Recently, Yuzhi Xu et al. demonstrated a significant inhibitory effect of a copper nanomaterial on bladder tumor growth in mice with negligible systemic toxicity (28). This suggests that selective killing of cancer cells by modulating the concentration of copper ions in cancer cells is a feasible and promising new direction for cancer therapy. However, as a novel form of RCD, the role of cuproptosis in PCa remains unclear.

In this study, we first visualized the expression, prognostic network, and somatic alteration of CRGs in the TCGA PCa cohort. Two molecular subtypes associated with cuproptosis were identified. Then, prognosis, clinicopathological features, function enrichment, TME, and immunotherapy response were compared between the two molecular subtypes. Next, on the basis of the differentially expressed genes (DEGs) between the two cuproptosis-related subtypes, we established and tested a prognostic signature consisting of five genes to evaluate prognosis independently for PCa in the TCGA database and validated the performance and generalizability of the signature in eight completely independent datasets. We also established a clinically applicable nomogram and analyzed the function enrichment, TME, somatic mutations, chemotherapy response prediction, and potential drug prediction on the basis of the risk signature. Finally, we validated the expression of model genes in cells, explored the regulation of these genes in the presence of copper ions and copper ionophore Elesclomol to induce cuproptosis, and further investigated the changes of CRGs at RNA and protein levels after knockdown of the key model gene B4GALNT4 by proteomics and transcriptomics analysis.

So far, the study of cuproptosis in PCa is still in its infancy. Our study explores this promising uncharted area in PCa and provides a reference for future research on cuproptosis in PCa.

## 2 Materials and methods

### 2.1 Data collection

This study included nine independent PCa cohorts (**Table 1**, **Supplementary Table S1**). Transcriptome profiles (Transcripts Per Kilobase Million, TPM) of 497 PCa cases and 52 normal cases were obtained from the Cancer Genome Atlas (TCGA) database (https://portal.gdc.cancer.gov/). The corresponding clinical and progression-free survival (PFS) information in TCGA were downloaded from the UCSC (University of California, Santa Cruz) Xena public data hub (https://xenabrowser.net/).

**Table 1:** Detailed information of PCa cohort used in this study.

Datasets	Platform	Number of Input(tumor)	Application
TCGA	Illumina HumanHT-12 V4.0 expression beadchip	497	Construction and Test of the Prognostic Signature
DKFZ	Illumina HumanHT-12 V3.0 expression beadchip	81	Validation of the Prognostic Signature
MSKCC	Affymetrix Human Exon 1.0 ST Array	140	Validation of the Prognostic Signature
CPGEA	Illumina HiSeq X TEN	125	Validation of the Prognostic Signature
GSE46602	GPL570 [HG-U133_Plus_2] Affymetrix Human Genome U133 Plus 2.0 Array	36	Validation of the Prognostic Signature
GSE70768	GPL10558 Illumina HumanHT-12 V4.0 expression beadchip	111	Validation of the Prognostic Signature
GSE70769	GPL10558 Illumina HumanHT-12 V4.0 expression beadchip	92	Validation of the Prognostic Signature
GSE70770	GPL10558 Illumina HumanHT-12 V4.0 expression beadchip	203	Validation of the Prognostic Signature
GSE54460	GPL11154 Illumina HiSeq 2000 (Homo sapiens)	91	Validation of the Prognostic Signature

Eight completely independent cohorts were included as the external validation sets, including DFKZ (The German Cancer Research Center, Deutsches Krebsforschungszentrum, n=81) (29), MSKCC (The Memorial Sloan Kettering Cancer Center, n = 140) (30), CPGEA (Chinese Prostate Cancer Genome and Epigenome Atlas, n=125) (31), GSE46602(n=36) (32), GSE70768 (n=111) (33), GSE70769 (n=92) (33), GSE70770 (n=203) (33), GSE54460 (n=91) (34). The cases included in the 8 external datasets were all radical surgery PCa cases with complete survival information. All 8 external datasets were used as validation sets only, and none of them were involved in the construction of the prediction model. The RNA sequence data profiles and the corresponding clinical information of DFKZ and MSKCC were obtained from the cBioPortal for Cancer Genomics (https://www.cbioportal.org/). The RNA sequence data of CPGEA were downloaded from (http://www.cpgea.com/download.php). Our team published the CPGEA dataset in Nature in 2020 (31), and we used the latest survival data in this study. The microarray data profiles and corresponding clinical information of GSE46602, GSE70768, GSE70769, GSE70770, and GSE54460 were obtained from Gene Expression Omnibus (GEO) database (https://www.ncbi.nlm.nih.gov/geo/).

We downloaded the complete expression data and detailed clinical information of the cohort of metastatic urothelial carcinoma treated with atezolizumab (an anti-PDL-1 agent) in a large phase 2 trial (IMvigor210) from the R package IMvigor210Core Biologies (version 1.0.0) (35). CRGs, including NFE2L2, NLRP3, ATP7B, ATP7A, SLC31A1, FDX1, LIAS, LIPT1, LIPT2, DLD, DLAT, PDHA1, PDHB, MTF1, GLS, CDKN2A, DBT, GCSH, and DLST, were obtained from the literature published in *Science* by Tsvetkov et al. (23).

### 2.2 Somatic mutation and copy number alteration analysis

We downloaded the somatic mutation data of PCa from the TCGA database and performed gene mutation waterfall plots through the “maftools” R package. Tumor mutation burden (TMB) was calculated for each patient, and differences in TMB were compared between different molecular subtypes and risk groups. Survival analysis was conducted based on the TMB score. We downloaded the somatic copy number alterations (SCNA) data of PCa from the UCSC Xena public data hub and compared the frequency of CRGs copy number gain and loss. And then, the somatic mutation frequencies of the model genes were exhibited using the cBioPortal database.

### 2.3 Consensus clustering analysis

Univariate cox regression analysis was conducted to screen out prognostic CRGs for PCa. Based on the expression of the prognostic CRGs, consensus clustering analysis was conducted with the R software package “ConsensusClusterPlus” to identify cuproptosis-related molecular subtypes. The Kaplan-Meier (K-M) analysis was used to compare the prognosis between the two groups. The correlation of clusters with CRGs and clinicopathological features was displayed by a heat map, and the differences in clinicopathological features between subtypes were compared by a chi-square test.

### 2.4 Gene set variation analysis and gene set enrichment analysis

Utilizing the “GSVA” R package, GSVA was performed to compare the differences in biological pathways between molecular subtypes. The adjusted p < 0.05 was used as the criterion for judging statistically significant differences in pathway enrichment among different subgroups by the “limma” package. The R package “clusterProfiler” was used to perform GSEA.

### 2.5 Immune landscape analysis

Each sample’s immune cell infiltration and functional activity were calculated using ssGSEA. Previous studies provided us with the marker genes of different immune cells (**Supplementary Table S2**) (36, 37). Immune, stromal, and estimate scores were calculated using the ESTIMATE algorithm based on the proportion of immune and stromal cells. We also compared the expression of major histocompatibility complex (MHC) and immune checkpoint molecules between subtypes and between the risk groups (38) and the expression of genes that inhibit the cancer-immunity cycle based on cluster analysis (39). These genes that inhibit the cancer-immunity cycle were downloaded from https://biocc.hrbmu.edu.cn/TIP/index.jsp . Tumor Immune Dysfunction and Exclusion (TIDE) score related to poorer immune checkpoint blockade therapy was calculated through the TIDE database.

### 2.6 Construction and validation of the prognostic signature

Firstly, we performed DEGs analysis between the two molecular subtypes by limma package in R software. The threshold for differential analysis was “Adjusted p<0.05 and | log2FoldChange| > 0.585”. Sixty-three prognostic DEGs for PCa were identified through univariate Cox regression analysis. Subsequently, we randomly divided 497 PCa patients from the TCGA cohort into a training group (n=249) and a test group (n=248). To eliminate potential overfitting between the prognostic DEGs, we used the least absolute shrinkage and selection operator (LASSO) algorithm with the penalty parameter (λ) determined by the lowest partial likelihood deviance based on the R package “glmnet” to establish a prognostic signature. The LASSO cox regression analysis with 10-fold cross-validation was conducted in the TCGA training group with the glmnet package in R to further select DEGs with the greatest predictive power. Finally, the forward stepwise selection and the multivariate cox regression model were utilized to develop a prognostic signature according to the candidate DEGs generated by the above screening. Then, the regression coefficients calculated by multivariate cox regression analysis were used to construct the cuproptosis-related risk score (CRRS).

According to the median risk score value of the training cohort, the TCGA cohort (including the training and test cohorts) was divided into high- and low-risk groups. The performance of the model was assessed using K-M analysis and area under the curve (AUC) of the receiver operating characteristic (ROC) curve. Furthermore, the reliability and generalizability of the model were validated by eight completely independent datasets (DFKZ, MSKCC, CPGSA, GSE46602, GSE70768, GSE70769, GSE70770, and GSE54460). Based on the model built from the training set in the TCGA dataset, risk scores for each patient in these external datasets were calculated separately. Then, patients in each external dataset were classified into high- and low-risk groups based on the optimal cutoff of risk scores calculated by the “surv_cutpoint” algorithm of the survminer R package. Finally, the progression-free survival time between the two groups was compared through K-M analysis and AUC of the ROC curve. In addition, we confirmed that CRRS is an independent prognostic factor for PCa using univariate and multivariate cox regression analyses and established a clinically applicable nomogram.

### 2.7 Chemotherapy response and small-molecule drugs

The response to chemotherapeutic drugs was predicted using the Genomics of Drug Sensitivity in Cancer (GDSC) database (37). The Half Maximal Inhibitory concentration (IC50) was calculated through the “pRRophetic” package (37).

The Connectivity Map (cMap) Database, a database of biological applications combining disease, gene expression, and small-molecule drugs, can predict compounds that may induce or reverse tumor biological processes by comparing up-and down-regulated genes between the two risk groups (37). Enrichment scores range from -100 to 0, indicating that these compounds may be potential candidates for PCa treatment. 3D structural maps of the six most likely candidates were obtained from the PubChem database (37).

### 2.8 Cell culture and drug therapy *in vitro*

Four PCa cell lines (C4-2, PC3m, PC3, and LNCaP) were used in this study, and these cell lines were purchased from the Cell Bank of the Chinese Academy of Science (Shanghai, China). All these cell lines were cultured in RPMI-1640 with 10% fetal bovine serum and 1% penicillin-streptomycin solution at 37°C in a humid incubator with 5% CO2. We purchased copper ionophore Elesclomol and copper chloride from Selleck and Sangon, respectively. The cells were treated with 2mM copper chloride or 20nM Elesclomol when the cells were adherent and morphologically diffused. After 24h of treatment, cells were collected, and RNA was isolated.

### 2.9 Real-time quantitative polymerase chain reaction

The total RNA of the above cells was isolated using the Fast Pure Cell Total RNA Isolation Kit (Vazyme, RC101-01). Then, reverse transcription was conducted with the HiScript III RT SuperMix for qPCR (+gDNA wiper) Kit (Vazyme, R323-01). Next, RT-qPCR was performed in triplicate with ChamQ Universal SYBR qPCR Master Mix (Vazyme, Q711). The mRNA expression level of B4GALNT, FAM83D, COL1A1, CHRM3, and MYBPC1 was normalized by β-actin mRNA. All experiments were conducted following the manufacturer’s protocol. The primer sequences are listed in **Table S3**.

### 2.10 Transfection of C4-2 cells with B4GALNT4-specific shRNA plasmid

The shRNA sequences for B4GALNT4 and the shRNA control were designed through GPP Web Portal (https://portals.broadinstitute.org/gpp/public/gene/search ). The sequences are also listed in **Table S3**. The lentivirus expression system was used to generate targeted virus supernatant for infection of C4-2 cells. After 48h of infection, the target cells were screened with puromycin. Then, western blotting confirmed the expression of B4GALNT4 in these target cells.

### 2.11 Western blot

The cells were lysed in RIPA (Radio Immunoprecipitation Assay) solution. After separation with 10% SDS-PAGE, the proteins were transferred to PVDF membranes and detected with antibodies. Anti-B4GALNT4 was purchased from Biorbyt (Cambridge, UK). Anti-GAPDH was purchased from ProteinTech (Chicago, USA). GAPDH was used as an internal reference.

### 2.12 4D label-free LC-MS/MS (liquid chromatography tandem-mass spectrometry) proteomics and data processing

We obtained samples from the C4-2 stable cell lines (shB4GALNT4 vs. shControl) by sonicating them three times on ice in lysis buffer (8 M urea, 1% protease inhibitor cocktail) with a high-intensity sonication processor (Scientz). BCA kits were used to measure the protein concentration of these samples after centrifugation at 12000 g for 10 minutes at 4°C. The following reduction with 5 mM dithiothreitol for 30 minutes at 56°C, the protein solution was alkylated for 15 minutes at room temperature with 11 mM iodoacetamide. Following that, 100 mM TEAB was added to the protein (urea concentration was below 2 M). Finally, the peptide was desalted by the C18 SPE column after digestion with trypsin. A reverse phase assay column (25 cm length, 75/100 mm internal diameter) was loaded directly with the tryptic peptide dissolved in solvent A (0.1% formic acid, 2% acetonitrile/water). For the separation of peptides, a gradient of 6% to 24% solvent B (0.1% formic acid in acetonitrile) was used for no less than 70 minutes, followed by a gradient of 24% to 35% in 14 minutes, 80% in 3 minutes, and 80% for the final 3 minutes. Peptides processed by capillary source were analyzed by timsTOF Pro (Bruker Daltonics) mass spectrometry (MS).

MaxQuant search engine (v.1.6.15.0) was used to process the obtained MS/MS data. The reverse decoy database was linked to the human SwissProt database (20422 entries) when searching tandem MS. Trypsin/P was designated as a lyase, allowing cleavage of up to 2 deletions. A mass tolerance of 20 ppm is set for the first precursor ion, five ppm for the main search, and 0.02 Da for the fragment ion. The false discovery rate (FDR) < 0.01 and Fold Change ≥1.2 were used to determine whether the expression differed significantly.

### 2.13 The transcriptomics analysis

Samples were obtained from the abovementioned C4-2 stable cell lines (shB4GALNT4 vs. shControl). Wash and dissolve the sample with 1 ml of TRizol reagent. With the help of a NanoPhotometer spectrophotometer (IMPLEN, California, USA), the purity of the RNA was determined. After the measurement of the concentration and integrity of RNA, the sequencing libraries were established with the NEBNext UltraTM RNA library Prep Kit for Illumina (NEB, USA). Then, based on the established libraries, paired-end reads were generated using the Illumina Hiseq 2500 platform. The depth of sequencing coverage was 10-fold, and the sequence read length was 200-250. Prior to data analysis, raw data was processed by eliminating reads with adapters, ploy-N, and low quality. The edgeR package was used to analyze the differential expression of two samples (without biological replicates). The threshold was the FDR < 0.01 and |log 2 (Fold Change) | ≥1.

### 2.14 Statistical analysis

All statistical analyses were performed using R software (version 4.2.0), except for the statistical analysis of qPCR results, which were analyzed by the analysis of variance (ANOVA) method based on GraphPad Prism (version 8.2.1). The differences between two cuproptosis-related molecular subtypes and two risk groups were analyzed through the Wilcoxon rank sum test. KM analysis was applied to compare PFS. Univariate and multivariate cox regression analyses were carried out to obtain independent predictors for PCa. It was considered statistically significant if the p-value was less than 0.05 (*, p < 0.05; **, p < 0.01; ***, p < 0.001).

## 3 Results

### 3.1 The expression, survival network and somatic alteration landscape of CRGs in TCGA cohort

**Figure 1:**
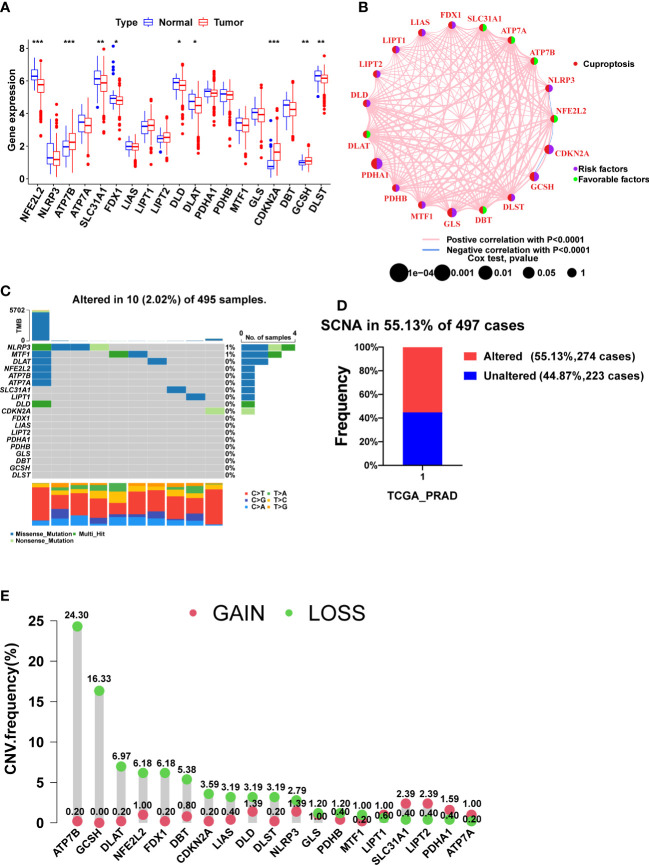
The expression, prognosis, and somatic alteration of CRGs in the TCGA PCa cohort. **(A)** The comparison of CRGs expression between tumor and normal tissues. **(B)** The PFS network of CRGs and co-expression relationship between CRGs in PCa. **(C)** The mutation frequency of CRGs in 495 PCa samples from the TCGA cohort. **(D)** Histogram of the SCNA frequency of CRGs in PCa. **(E)** Lollipop chart of the frequency of different SCNA types. (*, p < 0.05; **, p < 0.01; ***, p < 0.001).

Nine CRGs were differentially expressed between tumor and normal tissues, among which NFE2L2, SLC31A1, FDX1, DLD, DLAT, and DLST were lowly expressed in tumor tissues, and ATP7B, CDKN2A, and GCSH were highly expressed in tumor tissues (**Figure 1A**, p<0.05). Since FDX1, DLD, and DLAT are pro-cuproptosis genes while CDKN2A is an anti-cuproptosis gene (23), PCa may be in a state of suppression of cuproptosis.

**Figure 1B** shows the relationship between PCa prognosis and CRGs as well as the mutual co-expression relationship between these CRGs. The univariate cox regression analysis showed that PDHA1, GLS, CDKN2A, and GCSH were significantly associated with poor prognosis (**Figure 1B**, **Table 2**, p<0.05). All of the co-expression relationships between CRGs were positive except NLRP3 and NFE2L2, NFE2L2 and CDKN2A, and CDKN2A and GCSH, which were negative co-expression relationships (**Figure 1B**). KM analysis found that patients with high expression of PDHA1(p<0.001), GLS(p=0.002), LIPT1(p=0.002), CDKN2A(p=0.002), NLRP3 (p=0.011), GCSH (p=0.022), and DLST (p=0.023) had significantly shorter PFS time (**Supplementary Figures S1A-G**), while patients with high expression of NFE2L2 (p=0.003), DBT (p=0.007), SLC31A1 (p=0.021), ATP7A (p=0.028) and ATP7B (p=0.032) had significantly longer PFS time (**Supplementary Figures S1H-L**). In summary, there is a complex co-expression relationship between CRGs in prostate cancer, and almost all CRGs are positively regulated among themselves. Furthermore, CRGs were closely related to the prognosis of prostate cancer.

**Table 2:** The results of univariate Cox regression analysis and Kaplan–Meier survival analysis of CRGs in TCGA PCa cohort.

CRGs	HR	HR.95L	HR.95H	Unicox pvalue	KM pvalue
NFE2L2	0.832	0.625	1.107	0.207	0.003
NLRP3	1.166	0.863	1.576	0.318	0.011
ATP7B	0.899	0.693	1.166	0.421	0.032
ATP7A	0.938	0.713	1.233	0.644	0.028
SLC31A1	0.954	0.750	1.215	0.705	0.021
FDX1	1.379	0.828	2.296	0.217	0.063
LIAS	1.327	0.698	2.524	0.389	0.071
LIPT1	1.378	0.943	2.013	0.098	0.002
LIPT2	1.219	0.756	1.964	0.417	0.221
DLD	1.069	0.740	1.545	0.722	0.145
DLAT	0.977	0.761	1.255	0.858	0.102
PDHA1	2.583	1.379	4.840	0.003	<0.001
PDHB	1.072	0.679	1.692	0.767	0.338
MTF1	1.020	0.728	1.429	0.908	0.215
GLS	1.450	1.030	2.043	0.033	0.002
CDKN2A	1.289	1.008	1.649	0.043	0.002
DBT	0.794	0.593	1.065	0.123	0.007
GCSH	2.022	1.015	4.026	0.045	0.022
DLST	1.234	0.759	2.005	0.397	0.023

HR, Hazard ratio; Unicox, univariate Cox regression; KM, Kaplan–Meier curve analysis.

Remarkably, CRGs were rarely mutated in PCa patients (only 2.02%) (**Figure 1C**), but SCNA of CRGs occurred in more than 55% of PCa patients (**Figure 1D**). CRGs, except for LIPT1, SLC31A1, LIPT2, PDHA1, and ATP7A, have a higher frequency of copy number loss than gain, with ATP7B and GCSH having the highest frequency of copy number loss but almost no copy number gain (**Figure 1E**, **Supplementary Figure S2A**). Taken together, SCNA, not mutation, was found to be the main cause of dysregulation of CRGs in PCa.

### 3.2 Identification of cuproptosis-related molecular subtypes in PCa

**Figure 2:**
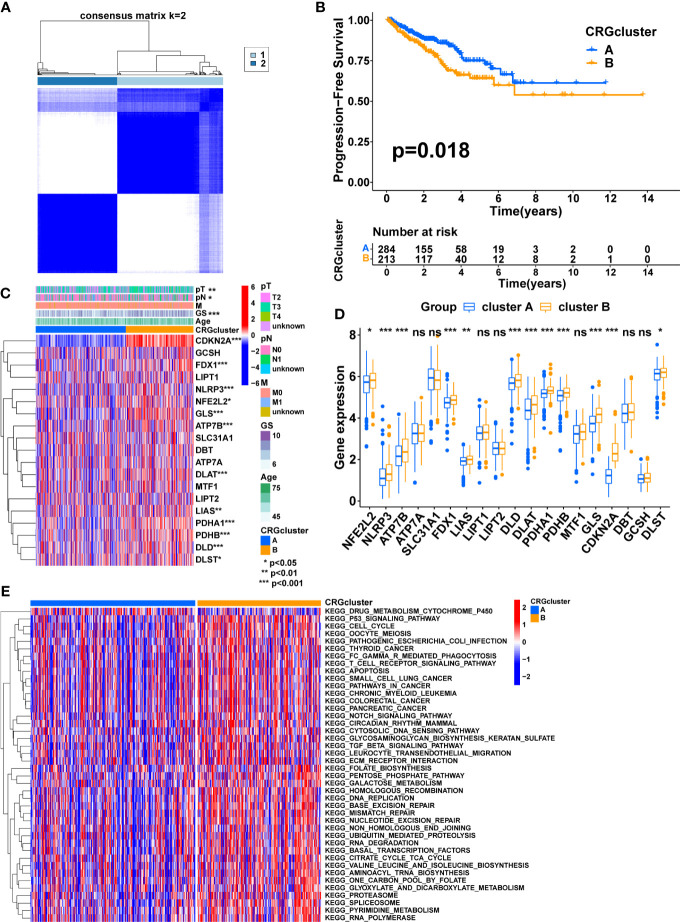
Consensus clustering of CRGs in PCa. **(A)** Consensus clustering matrix when k = 2. **(B)** The difference in PFS between the two clusters. **(C)** The heatmap shows the expression of CRGs between the two clusters and the correlations between the clusters and clinical features. **(D)** The comparison of CRGs expression between the two clusters. **(E)** The heatmap shows the result of GSVA between the two clusters. TNM, tumor node metastasis; p, pathology; GS, Gleason score. (*, p < 0.05; **, p < 0.01; ***, p < 0.001). ns, no significant.

Four prognostic CRGs were identified through univariate cox regression analysis (**Figure 1B**, **Table 2**, p<0.05). Based on the expression levels of these CRGs, an unsupervised clustering approach was carried out to classify 497 PCa patients from the TCGA cohort into two cuproptosis-related subtypes, with 284 cases in cluster A and 213 cases in cluster B (**Figure 2A**, **Supplementary Figures S2B-M**). KM analysis indicated that cluster B had a poorer prognosis (**Figure 2B** p=0.018). Next, we compared the expression of CRGs and the distribution of clinical features between the two subtypes (**Figure 2C**). There were twelve CRGs differentially expressed across the two subtypes, and all were highly expressed in cluster B (**Figure 2D**, p<0.05). There was a difference in clinical characteristics between the two subtypes in terms of Gleason score, pathological T-stage, and pathological N-stage, with cluster B showing a higher proportion of patients with a high Gleason score (p<0.001), high pathological T-stage (p<0.01) and high pathological N-stage (p<0.05) (**Table 3**). In summary, CRGs can divide PCa into two subtypes with completely different prognostic and clinical characteristics.

**Table 3:** The distribution of clinical features of PCa patients between the two clusters.

Characteristics	N (%) Entire dataset (n=497)	N (%)		*P*
		Cluster A (n=284)	Cluster B (n=213)	
Age, years				0.2965
<=65	354(71.23)	208(73.24)	146(68.54)	
>65	143(28.77)	76(26.76)	67(31.46)	
Gleason score				0.0001
6	45(9.05)	34(11.97)	11(5.16)	
7	247(49.70)	156(54.93)	91(42.72)	
8	64(12.88)	36(12.68)	28(13.15)	
9	137(27.57)	56(19.72)	81(38.03)	
10	4(0.80)	2(0.70)	2(0.94)	
pT stage				0.0019
T2	187(37.63)	126(44.37)	61(28.64)	
T3	293(58.95)	152(53.52)	141(66.20)	
T4	10(2.01)	3(1.06)	7(3.29)	
unknown	7(1.41)	3(1.06)	4(1.88)	
pN stage				0.0201
N0	345(69.42)	208(73.24)	137(64.32)	
N1	79(15.90)	34(11.97)	45(21.13)	
unknown	73(14.69)	42(14.79)	31(14.55)	
M stage				0.1312
M0	455(91.55)	261(91.90)	194(91.08)	
M1	3(0.60)	0(0.00)	3(1.41)	
unknown	39(7.85)	23(8.10)	16(7.51)	

PCa, Prostate cancer; TNM, tumor node metastasis; p, pathology.

According to these results, CRGs may be involved in tumor development *via* some underlying mechanisms. Therefore, GSVA was performed to explore the potential mechanisms. The result showed that most of the pathways involved in metabolism, immunity, and cancer, including the TCA cycle, FC gamma R-mediated phagocytosis, Leukocyte transendothelial migration, T cell receptor signaling pathway, P53 signaling pathway, pathways in cancer, Notch signaling pathway, TGF beta signaling pathway, and ECM-receptor interaction, were significantly enriched in cluster B, which may contribute to the poorer prognosis (**Figure 2E**, **Supplementary Figure S2N**).

### 3.3 The immune-related characteristics of cuproptosis-related subtypes

**Figure 3:**
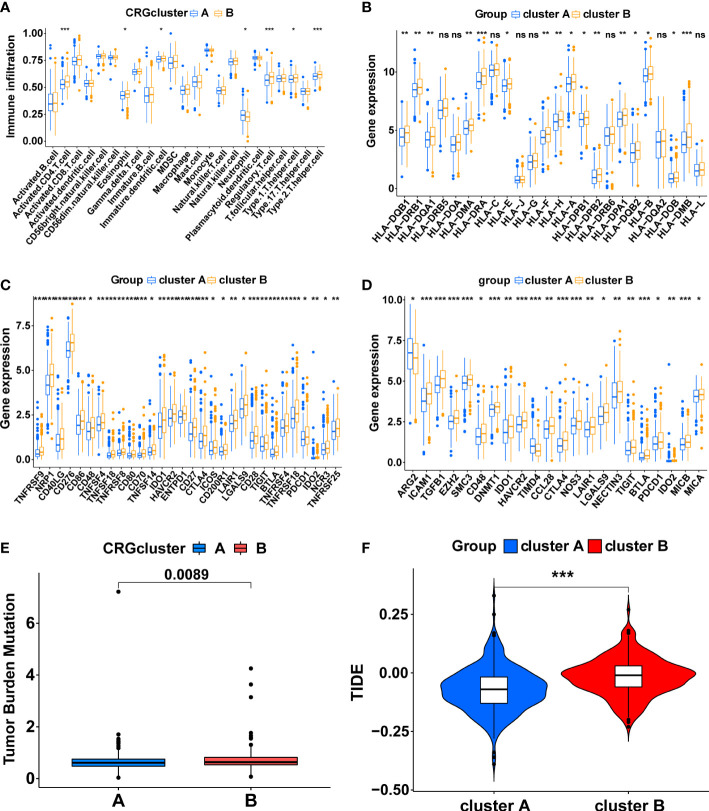
The immune-related characteristics of cuproptosis-related molecular subtypes in the TCGA cohort. **(A)** The difference in immune cell infiltration between the two clusters. **(B)** The comparison of MHC molecules expression between the two clusters. **(C)** Immune checkpoint molecules expression between the two clusters. **(D)** The expression level of the genes that inhibit the cancer-immunity cycle between the two clusters. The comparison of the TMB score **(E)** and TIDE score **(F)** between the two clusters. (*, p < 0.05; **, p < 0.01; ***, p < 0.001). ns, no significant.

The ssGSEA algorithm was utilized to compare immune infiltration between the two subtypes. The high infiltration of Neutrophils characterized cluster A, whereas cluster B was characterized by the high infiltration of Activated CD4 T cells, Eosinophil, Immature dendritic cells, Regulatory T cells, Type 1 T helper cells, and Type 2 T helper cells (**Figure 3A**, p<0.05). Furthermore, the expression of MHC molecules between the two subtypes was compared. The expression levels of MHC molecules were higher in cluster B except for HLA-DRB5, HLA-DOA, HLA-C, HLA-J, HLA-G, HLA-DRB6, HLA-DQA2 and HLA-L (**Figure 3B**, p <0.05).

Subsequently, a series of evaluation indicators were used to determine whether cuproptosis-related subtypes were significantly associated with immunotherapy effects, including the expression of immune checkpoint molecules and genes that inhibit cancer-immunity cycles, TMB scores, and TIDE scores. There were 30 differentially expressed immune checkpoint molecules between subtypes. All of them were highly expressed in cluster B (p<0.05), including PD-1 (PDCD1), CTLA4, B7H3 (CD276), HAVCR2, and TIGIT (**Figure 3C**, **Supplementary Figures S3A, B**). 22 genes that inhibit the cancer-immunity cycle were differentially expressed between subtypes (p<0.05), and all of them, except ARG2 and TIMD4, were significantly overexpressed in cluster B (**Figure 3D**, **Supplementary Figures S3C, D**). Meanwhile, cluster B had a higher TMB score (**Figure 3E**, p<0.01) and TIDE score (**Figure 3F**, p<0.001). In summary, These results show a complex immune microenvironment for the different subtypes, with cluster B appearing to exhibit a more suppressed immune microenvironment.

### 3.4 Construction and validation of a cuproptosis-related signature

**Figure 4:**
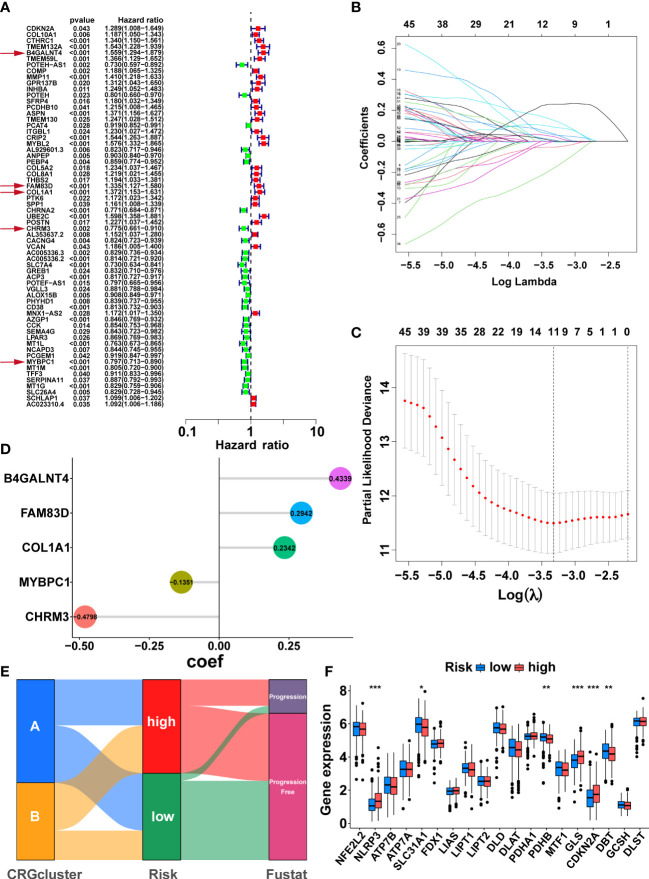
Development of the cuproptosis-related signature in the TCGA training cohort. **(A)** Sixty-three prognosis-related DEGs were identified by univariate Cox regression. The genes indicated by red arrows are the five genes involved in the construction of the prognostic model. **(B)** The horizontal axis represents the logarithm of the independent variable λ, and its coefficients are shown on the vertical axis. **(C)** The confidence interval corresponds to each lambda. **(D)** Coefficients of the five prognostic genes in the model. **(E)** Sankey diagrams displayed the correlation between cuproptosis-related subtypes, risk score, and prognosis. **(F)** The comparison of the expression levels of CRGs between two risk groups. (*, p < 0.05; **, p < 0.01; ***, p < 0.001).

Firstly, 147 DEGs between the two cuproptosis-related subtypes were identified by differential analysis (**Supplementary Figure S3E**). Next, 63 DEGs associated with PFS were obtained *via* univariate cox regression (**Figure 4A**, P<0.05). Subsequently, we randomly divided 497 PCa patients from the TCGA cohort into a training group (n=249) and a validation group (n=248), and there was no significant difference in clinicopathological features between the two groups (**Supplementary table S4**, P>0.05). In the training group, we further screened the optimal prognostic biomarkers by LASSO regression analysis, and 11 DEGs were selected with 10-fold cross-validation (**Figures 4B, C**). Then, the model with the lowest Akaike information criterion (AIC) value was established through multivariate cox regression analysis. Finally, we generated a risk score model consisting of five DEGs, including B4GALNT4, FAM83D, COL1A1, CHRM3, and MYBPC1. Forest plots showed the association of expression levels of the five model genes with PFS, where B4GALNT4 had the most considerable contribution to poorer prognosis (**Figure 4A**, Hazard ratio=1.559, p<0.01). The coefficient of each gene in the signature was exhibited in **Figure 4D**, and the risk score was calculated with the equation: CRRS=(0.4339∗B4GALNT4)+(0.2942∗FAM83D)+(0.2342∗COL1A1)+(−0.1351∗MYBPC1)+ (−0.4798∗CHRM3). The median risk score value of the training cohort was utilized to classify patients into high- and low-risk groups in the TCGA cohort.

Sankey diagrams illustrated the correlation between cuproptosis-related subtypes, risk score, and prognosis, and the patients with disease progression mainly were from the high-risk group (**Figure 4E**). The comparison of CRGs expression between the two risk groups is exhibited in **Figure 4F**. NLRP3, GLS, and CDKN2A were highly expressed in the high-risk group, while SLC31A1, PDHB, and DBT were lowly expressed in the high-risk group (**Figure 4F**, p<0.05). As expected, cluster A, with the better prognosis among the cuproptosis-related subtypes, had a lower risk score (**Supplementary Figure S3F**, p<0.001).

**Figure 5:**
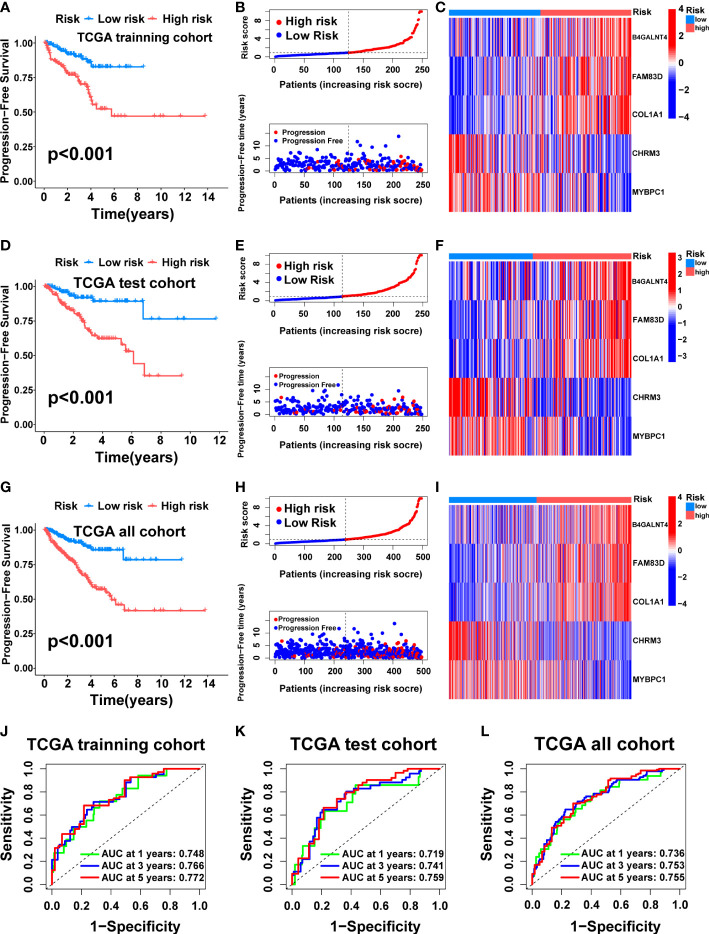
Construction and internal validation of the cuproptosis-related signature. For the TCGA training cohort: Kaplan–Meier curve **(A)**, risk score and survival status **(B)**, the expression heat map of the 5 model genes **(C)**, ROC curve, and AUC of the 5-gene signature **(J)**. For the TCGA test cohort: Kaplan–Meier curve **(D)**, risk score and survival status **(E)**, the expression heat map of the 5 model genes **(F)**, ROC curve, and AUC of 5-gene signature **(K)**. For the TCGA all cohort: Kaplan–Meier curve **(G)**, risk score and survival status **(H)**, the expression heat map of the 5 model genes **(I)**, ROC curve, and AUC of the 5-gene signature **(L)**.

Then, we tested the performance of the signature in the TCGA cohort. KM analysis suggested that the high-risk patients had poorer PFS than the low-risk patients in the TCGA training (**Figure 5A**, p<0.001), test (**Figure 5D**, p<0.001), and all (**Figure 5G**, p<0.001) cohorts. We also visualized the risk score distribution and survival status in these cohorts. The results showed that a higher risk score was associated with a higher risk of disease progression and a shorter PFS period (**Figures 5B, E, H**). The model genes in the three cohorts also exhibited a similar expression pattern (**Figures 5C, F, I**). Then, the ROC curve was used to assess the performance of the signature. In the TCGA training cohort, the mean AUC values for predicting 1-, 3-and 5-year prognosis were 0.748, 0.766, and 0.772, respectively (**Figure 5J**). As for the TCGA test cohort, the average AUC values for 1-, 3- and 5-year prognostic prediction were 0.719, 0.741, and 0.759, respectively (**Figure 5K**). In addition, the mean AUC values for predicting 1-, 3- and 5-year PFS were 0.736, 0.753, and 0.755 in the entire TCGA cohort (**Figure 5L**).

**Figure 6:**
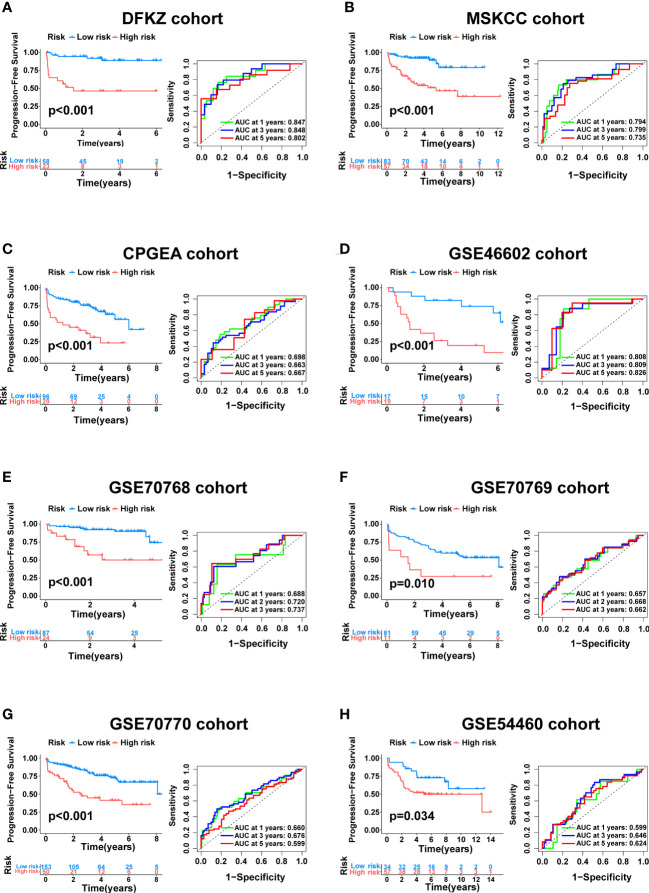
External validation of the cuproptosis-related signature. Kaplan–Meier curve as well as ROC curve and AUC of the signature in DFKZ cohort **(A)**, MSKCC cohort **(B)**, CPGEA cohort **(C)**, GSE46602 cohort **(D)**, GSE70768 cohort **(E)**, GSE70769 cohort **(F)**, GSE70770 cohort **(G)** and GSE54460 cohort **(H)**.

To further verify the generalizability of the signature, external validation was performed on eight completely independent datasets (DFKZ, MSKCC, CPGEA, GSE46602, GSE70768, GSE70769, GSE70770, and GSE54460), in which the CPGEA dataset was published in *Nature* by our team in 2020 (31), and we used the latest follow-up data in this study. Consistently, patients in the low-risk group had significantly longer PFS time in the eight cohorts, including the DFKZ cohort (n=81, p<0.001, **Figure 6A**), the MSKCC cohort (n=140, p<0.001, **Figure 6B**), the CPGEA cohort (n=125, p<0.001, **Figure 6C**), the GSE46602 cohort (n=36, p<0.001, **Figure 6D**), the GSE70768 cohort (n=111, p<0.001, **Figure 6E**), the GSE70769 cohort (n=92, p=0.01, **Figure 6F**), the GSE70770 cohort (n=203, p<0.001, **Figure 6G**), and the GSE54460 cohort (n=91, p=0.034, **Figure 6H**). Furthermore, the ROC curves demonstrated the good predictive performance of the signature in these datasets (**Figure 6**). In summary, this signature has good generalizability and application prospects.

**Figure 7:**
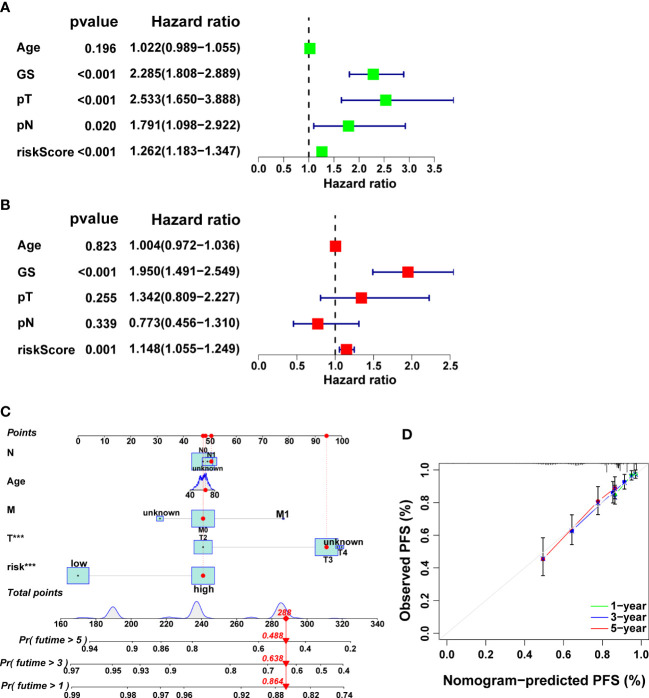
Independent prognostic analysis as well as the development and validation of a nomogram in the TCGA cohort. The results of univariate **(A)** and multivariate **(B)** Cox regression analysis. **(C)** The nomogram for predicting PFS in PCa. **(D)** Calibration plots of the nomogram.

Remarkably, it was verified that CRRS is an independent prognostic factor for PCa through univariate and multivariate cox regression analysis (**Figures 7A-B**, p<0.01). Finally, we developed a clinically applicable nomogram to predict 1-, 3-, and 5-year prognosis for PCa patients (**Figure 7C**). The calibration curves illustrated good consistency between actual 1-, 3- and 5-year PFS rates and predicted PFS rates (**Figure 7D**).

### 3.5 The immune landscape of the signature

**Figure 8:**
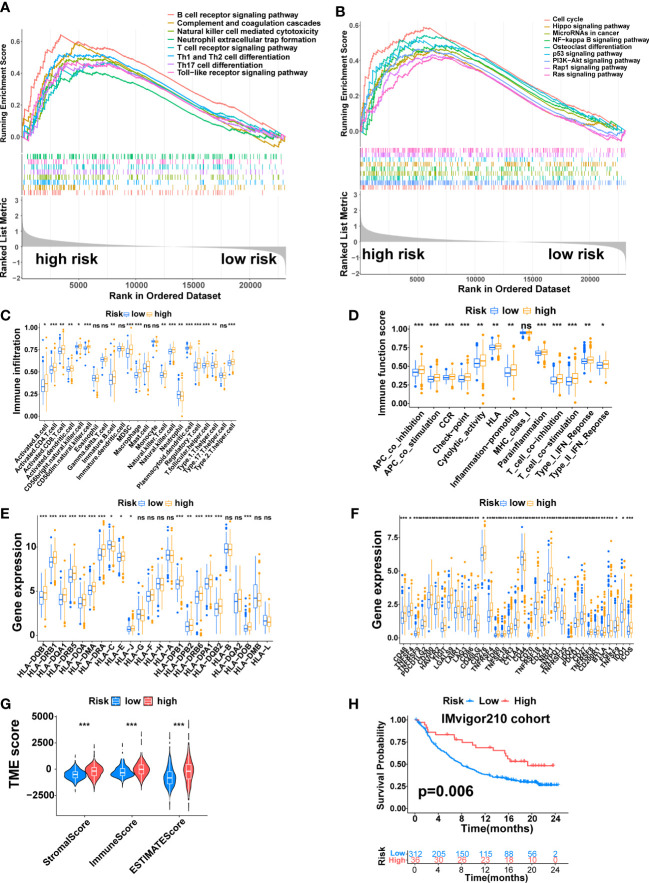
The Immune Landscape of the Signature. **(A)** Immune-related pathways enriched in the high-risk group. **(B)** Tumor-related pathways enriched in the high-risk group. **(C)** The difference in immune cell infiltration between the two risk groups. **(D)** The difference in immune-related functions or pathways between the two risk groups. **(E)** The comparison of MHC molecules expression between the two risk groups. **(F)** Immune checkpoint molecules expression between the two risk groups. **(G)** Stromal score, immune score, and estimate score between the two risk groups. **(H)** K-M analysis of the IMvigor210 cohort. (*, p < 0.05; **, p < 0.01; ***, p < 0.001). ns, no significant.

Previous studies have shown that tumor immune microenvironments are essential for tumor development (40, 41). Consequently, to explore the causes of poorer prognosis in the high-risk group, GSEA was conducted to investigate the enrichment of immune-related pathways and tumor-related pathways in the group. The result showed that many immune-related pathways were enriched in the high-risk group, including the B cell receptor signaling pathway, Natural killer cell mediated cytotoxicity, Neutrophil extracellular trap formation, T cell receptor signaling pathway, Th1 and Th2 cell differentiation, Th17 cell differentiation, and Toll−like receptor signaling pathway (**Figure 8A**). We also found that several classic tumor-related pathways were enriched in the high-risk group, including the Hippo signaling pathway, NF-kappa B signaling pathway, p53 signaling pathway, PI3K-Akt signaling pathway, Rap1 signaling pathway, and Ras signaling pathway (**Figure 8B**).

Next, the correlation between this signature and the tumor immune microenvironment was further explored. The ssGSEA algorithm revealed higher immune cell infiltration and more active immune-related functions in the high-risk group. The immune cells that differentially infiltrated between the two risk groups were more infiltrated in the high-risk group except for Neutrophi, which was more infiltrated in the low-risk group (**Figure 8C**, p<0.05). The twelve immune-related functions that were differentially enriched between the two risk groups were all more active in the high-risk group (**Figure 8D**, p<0.05). Next, we explored the expression of MHC molecules and found that sixteen MHC molecules were differentially expressed between the two risk groups. Except for HLA-C, which was highly expressed in the low-risk group, the rest were highly expressed in the high-risk group (**Figure 8E**, p<0.05). Furthermore, the expression of immune checkpoint molecules and genes that inhibit the cancer-immunity cycle was also explored. A total of 35 immune checkpoint molecules were differentially expressed between the two risk groups. Except for CD44 and FGL1, which were highly expressed in the low-risk group, the rest were highly expressed in the high-risk group, including PD-1 (PDCD1), PDL1 (CD274), CTLA4, HAVCR2, B7H3(CD276), TIGIT and LAG3 (**Figure 8F**, p<0.05).

Finally, we compared the TME between the two risk groups through the ESTIMATE algorithm. The result revealed higher immune, stromal, and ESTIMATE estimation scores in the high-risk group (**Figure 8G**, p<0.001). On the IMvigor210 cohort, we performed a K-M analysis to assess the value of this signature in predicting immune response to immunotherapy, which revealed that high-risk patients had a longer OS time than low-risk patients (**Figure 8H**, p=0.006).

### 3.6 Somatic mutation and TMB of the signature

**Figure 9:**
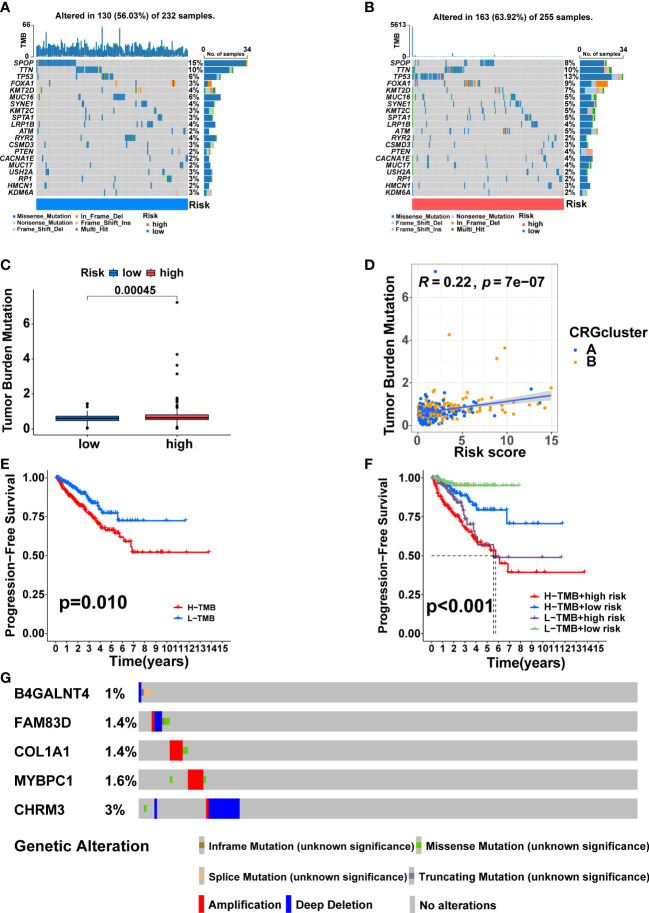
Somatic mutation and TMB based on the signature. Waterfall maps of the somatic mutations in the low-risk group **(A)** and the high-risk group **(B)**. **(C)** Difference of TMB between the two risk groups. **(D)** Correlation between risk score and TMB. **(E)** Comparison in PFS between high- and low-TMB groups. **(F)** Comparison in PFS based on TMB and risk score. **(G)** Mutation frequencies of the five model genes in PCa patients from the cBioPortal database.

SPOP (15%), TTN (10%), TP53 (6%), FOXA1 (3%), and KMT2D (4%) accounted for the highest mutation frequencies in the low-risk group, while SPOP (8%), TTN (10%), TP53(13%), FOXA1 (9%) and KMT2D (7%) had the highest mutation frequencies in the high-risk group (**Figures 9A, B**). Furthermore, the difference in TMB between the two risk groups was also compared. The high-risk group had higher TMB (**Figure 9C**, p<0.001), and TMB was positively correlated with risk score (**Figure 9D**, R = 0.22, p = 7e−07). KM analysis showed a shorter duration of PFS in patients with high TMB (**Figure 9E**, p<0.05). After combining with the signature, the prognosis of the high TMB + high-risk group was significantly poorer than that of the low TMB + low-risk group (**Figure 9F**, p<0.001). Finally, we found that the mutation frequencies of the five model genes were all low in PCa (**Figure 9G**).

### 3.7 Predicting chemotherapy response and screening small molecule drug

**Figure 10:**
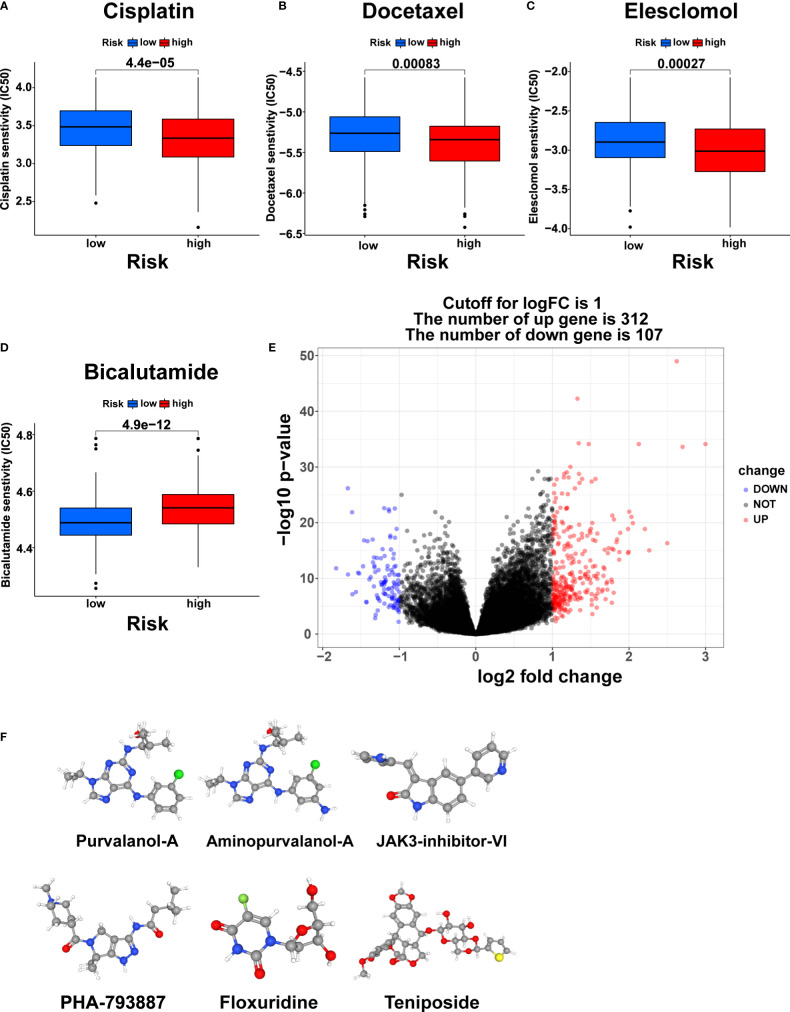
Chemotherapy response prediction and small molecule drug screening. The differences in the chemotherapy response of Cisplatin **(A)**, Docetaxel **(B)**, Elesclomol **(C)**, and Bicalutamide **(D)** between the two risk groups. **(E)** Volcano plot of DEGs between the two risk groups. **(F)** The 3D structure of six small molecule drugs screened out from the cMap database. IC50, the half maximal inhibitory concentration.

The differences in response to commonly used chemotherapeutic drugs between the two risk groups from TCGA were predicted *via* the GDSC dataset. We identified 53 chemotherapeutic drugs with significantly different IC50 values between the two risk groups, including 45 drugs with lower IC50 values in the high-risk group and 8 drugs with lower IC50 values in the low-risk group (**Supplementary Table S5**, **Supplementary Figures S4**, **S5**, p<0.001). Remarkably, the three most commonly used chemotherapy agents (Cisplatin, Docetaxel, and Bicalutamide) in PCa treatment and the copper ionophore Elesclomol that can induce cuproptosis showed significant differences in IC50 values between the two risk groups (23). Cisplatin, Docetaxel, and Eleclomol had lower IC50 values in the high-risk group, while Bicalutamide had a lower IC50 value in the low-risk group (**Figures 10A-D**, p<0.001).

Furthermore, we screened small-molecule drugs through the cMap database to identify potential treatment candidates for PCa patients. Based on the 312 upregulated genes and 107 downregulated genes generated by differential expression analysis between the two risk groups (**Figure 10E**, |logFC|>1, pvalue<0.05), the six most relevant small-molecule drugs (Purvalanol-A, Aminopurvalanol-A, JAK3-inhibitor-VI, PHA-793887, Floxuridine, and Teniposide) were screened out. Their 3D structures were exhibited *via* the PubChem database (**Figure 10F**).

### 3.8 The expression and regulation of model genes in cell lines and the further experiment on B4GALNT4

**Figure 11:**
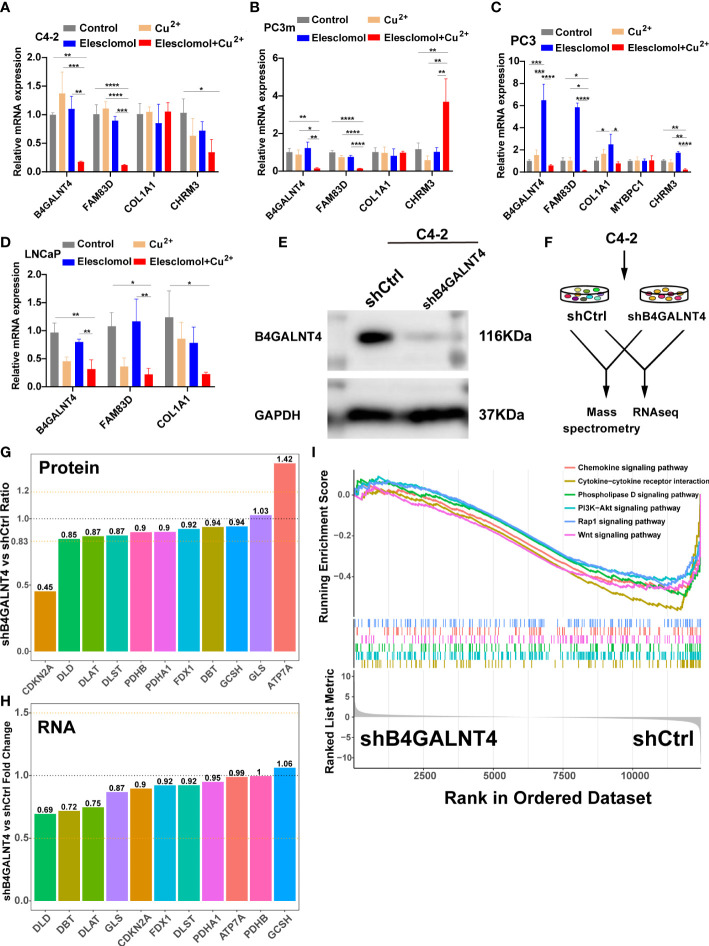
The expression and regulation of these model genes and further experiments on B4GALNT4. **(A–D)** qRT-PCR shows the expression and regulation of model genes in prostate cell lines treated with drugs that induce cuproptosis for 24 h (n = 3). CuCl2 (2mM), Elesclomol (20 nM), both CuCl2 (2mM) and Elesclomol (20 nM). **(E)** Western blot showing the knockdown effect of B4GALNT4 in C4-2. **(F)** Experimental scheme of proteomics and transcriptomics analysis on C4-2 stable cell lines with B4GALNT4 knockdown. **(G)** The changes in protein levels of CRGs after B4GALNT4 knockdown. **(H)** The changes in mRNA levels of CRGs after B4GALNT4 knockdown. NS, P >= 0.05; *, P < 0.05; **, P < 0.01; ***, P < 0.001; ****, P < 0.0001. **(I)** GSEA demonstrated the enrichment of tumor-related pathways after B4GALNT4 knockdown.

To validate the results of the above analysis, the mRNA expression of the five model genes and the regulation of these genes in the presence of copper ions and copper ionophore Elesclomol were explored by qRT-PCR in various PCa cell lines (C4-2, PC3m, PC3, LNCaP). The results showed that B4GALNT4, FAM83D, COL1A1, and CHRM3 were stably expressed in the majority of PCa cell lines, while MYBPC1 was detected only in PC3 (**Figures 11A–D**). In addition, most of the model genes showed varying degrees of downregulation in the presence of Cu^2+^ and Elesclomol in most PCa cell lines, with B4GALNT4 and FAM83D being the most significant (**Figures 11A-D**, p<0.05), demonstrating the close association of these two genes with cuproptosis in prostate cancer cells.

Considering that B4GALNT4 contributed the most to poor prognosis, we conducted further research on B4GALNT4. Since B4GALNT4 has a high expression level in the C4-2 cell line, we constructed a stably transfected C4-2 cell line with the knockdown of B4GALNT4 (**Figure 11E**). Next, we performed proteomics and transcriptomics analyses using these stably transfected C4-2 cells (**Figure 11F**). In proteomics analysis, the CDKN2A protein level was significantly upregulated (Ratio<0.83), and the ATP7A protein level was significantly downregulated (Ratio>1.2) (**Figure 11G**). However, the transcriptomics analysis suggested that the RNA levels of these CRGs did not change significantly after the knockdown of B4GALNT4 (|logFoldChange|<1, **Figure 11H**). Additionally, GSEA analysis revealed that multiple cancer-related pathways were inhibited after the knockdown of B4GALNT4, including the PI3K−Akt signaling pathway, Rap1 signaling pathway, and Wnt signaling pathway. (**Figure 11I**). In summary, these results suggested that B4GALNT4 is a potential cuproptosis-related oncogene in PCa, which could be used as a target to treat PCa in combination with cuproptosis.

## 4 Discussion

Growing evidence suggests that genetic biomarkers have become increasingly crucial in highly personalized precision medicine (42). As tumor molecular biology advances, developing new predictive tools and therapeutic targets based on prognosis-related genes has become a promising field. These genes reflecting tumor progression at the molecular level not only contribute to more accurate personalized survival prediction and guide the choice of treatment regimens, but also help to develop molecular targets for precision treatment.

Cuproptosis, a newly discovered RCD form dependent on mitochondrial respiration, differs from any known RCD form (23). As a novel RCD form, cuproptosis has rapidly become a research hotspot, providing additional references for drug development and refinement of clinical indicators (15). Current studies have shown that cuproptosis is associated with prognosis and TME in bladder, breast, and hepatocellular carcinoma, and a series of good prognostic models have been developed to predict tumor prognosis (24, 25, 27). However, in PCa, studies related to cuproptosis are still in the preliminary stage and most of them have focused on cuproptosis-related long non-coding RNA (lncRNA). Several studies have now reported that cuproptosis-related lncRNA have a better prognostic role in predicting PCa (43, 44). However, studies on cuproptosis-related coding genes in PCa are rarely reported, and the role of cuproptosis in PCa remains unknown.

In this study, PCa can be stratified into two molecular subtypes according to the expression of prognostic CRGs. The prognosis of the two subtypes was significantly different, and the PFS time of cluster A was significantly longer than that of cluster B. Twelve CRGs were highly expressed in cluster B, among which CDKN2A was the most significant. As an anti-cuproptosis gene, the significantly high expression of CDKN2A in cluster B may indicate an inhibitory state of cuproptosis in cluster B (23). In addition, the analysis of clinicopathological features showed that cluster B had more advanced and malignant PCa cases. These results may explain the poorer prognosis of cluster B to some extent. Furthermore, we explored the underlying causes of these differences between the two clusters through GSVA. The result showed that the TCA cycle was significantly enriched in cluster B, which is enlightening considering the pivotal role of the TCA cycle in the process of cuproptosis.

The TME is a critical component of the growth of tumors. It comprises several types of cells, including tumor cells, infiltrating immune cells, and stromal cells. Tumor progression depends heavily on the crosstalk between these cells and between these cells and other non-cellular components (45). It has been revealed that the infiltration of different immune cells is closely associated with clinical outcomes of breast cancer, bladder cancer, and PCa (46–48). Therefore, the TME between cuproptosis-related subtypes was further compared. Patients in cluster B exhibited higher infiltration of immunosuppressive components, such as regulatory T (Treg) cells and activated CD4 T cells, whereas there was no difference in the proportion of anti-tumor immune cells, such as CD8 T cells and B cells, between the two clusters. Tumor-infiltrating Treg cells can inhibit anti-tumor immunity and promote cancer progression, which can cause adverse clinical outcomes, so it is considered the main obstacle to the successful application of immunotherapy (49–51). The recruitment and activation of CD4+ T lymphocytes are related to establishing a tumor immunosuppressive microenvironment (52). These previous findings suggest a tumor-promoting and anti-immune state in cluster B. Furthermore, most of the immune checkpoint genes (including PD-1 and CTLA4) and genes that inhibit the cancer‐immunity cycle were also highly expressed in cluster B, which further indicated the immunosuppressive state in cluster B. PD-1 and CTLA4 were highly expressed in cluster B, suggesting that patients in cluster B may benefit more from anti-PD1/CTALA4 therapy. However, as indicated by TIDE analysis, anti-PD1/CTALA4 therapy was less effective in cluster B, which reflects the complexity of the TME and requires more in-depth research to elucidate the interactions between the various cellular and matrix components.

Although significant progress has been made in diagnosing and treating PCa in recent decades, PCa is currently the second leading cause of cancer death in Western countries (53). Lack of accurate prognostic prediction tools and drug resistance are two significant challenges in PCa treatment (54). The accurate prognostic prediction could determine whether patients benefit from more aggressive therapies, including neoadjuvant therapy, more intensive surgery, chemotherapy, radiotherapy, targeted therapy, and immunotherapy, which could be customized for individual patients to improve outcomes. Therefore, we established and tested a prognostic signature in this study to independently evaluate PCa patients’ prognoses. The reliability and generalizability of the signature were verified in eight completely independent datasets involving a total of 879 PCa patients from multiple centers. Furthermore, a clinically applicable nomogram with high reliability for clinical practice was established. Interestingly, we found that pro-cuproptosis genes such as PDHB and SLC31A1 were lowly expressed in the high-risk group, while anti-cuproptosis genes such as GLS and CDKN2A were highly expressed in the high-risk group (23), indicating that PCa patients with high CRRS may be in an inhibited state of cuproptosis.

To further explore the mechanisms underlying the difference in prognosis between the two risk groups, we visualized pathway enrichment and immune landscape between the two groups. GSEA revealed that several classical cancer-related pathways, including the Hippo signaling pathway, NF-Kappa B signaling pathway, PI3K-Akt signaling pathway, and Ras signaling pathway, were enriched in the high-risk group. Among them, the Hippo signaling pathway and NF-Kappa B signaling pathway can promote metastasis and castration resistance of PCa (55–58). Studies have shown that the PI3K-Akt signaling pathway can interact with multiple cellular signaling cascades to promote PCa progression and influence ADT sensitivity in PCa cells (59). The interaction of the Ras signaling pathway and the Wnt signaling pathway can promote bone metastasis of PCa (60). Remarkably, several pro-tumor immune pathways, including T cell receptor signaling pathway, B cell receptor signaling pathway, Natural killer cell mediated cytotoxicity, Neutrophil extracellular trap formation, Th17 cell differentiation, and Toll−like receptor signaling pathway were also enriched in the high-risk group (61–63). These results explain, to some extent, the worse prognosis of the high-risk group.

Currently, immunotherapy has revolutionized the treatment strategy for many types of cancer (64, 65). However, due to the immune “cold” status of advanced PCa, which is usually characterized by poor T-cell infiltration, low mutational load, low MHC class I expression, and low PD-L1 expression (66, 67), the overall efficacy of single immunotherapy in cold tumors, including PCa, is poor (68, 69). In fact, PCa, as an indolent tumor, is an ideal model for cancer immunotherapy because it can provide sufficient time to form the anti-tumor immune response. With the approval of Sipuleucel-T for PCa treatment, tumor immunotherapy has achieved good efficacy in carefully selected PCa patients (70). In addition, combining tumor immunotherapy with ADT, chemotherapy, or DNA-damaging treatment can significantly promote the effect of immunotherapy, reflecting the excellent prospect of immunotherapy in treating PCa (70, 71). Therefore, apart from finding the optimum treatment combination, there is an urgent need to develop biomarkers that can predict tumor immune microenvironment and immunotherapy response, which are essential for the personalized treatment of patients with advanced PCa. Since the immune environment of TME is crucial for effective immunotherapy, we visualized the immune landscape of the two risk groups. Overall, patients in the high-risk group had higher immune cell infiltration, more active immune-related functions, higher expression of MHC molecules and immune checkpoint molecules (including PDL-1, PD-1, CTLA4, HAVCR2, B7H3, TIGIT, and LAG3), and higher immune scores. According to these findings, high-risk patients may experience a stronger immune response to tumor progression and may benefit more from immune checkpoint inhibitors (ICIs). Considering that high-risk patients have a higher TMB and that the immune system readily recognizes and kills tumor cells with high genomic instability (72), this again suggests that these patients may benefit more from immunotherapy. To further validate the role of CRRS in predicting the response to immunotherapy, we performed a K-M analysis on the IMvigor210 cohort. As expected, patients in the high-risk group had longer OS than those in the low-risk group. Thus, CRRS may help to screen patients who may benefit more from immunotherapy.

Chemotherapy is a significant treatment for advanced PCa. It is of great importance to choose a suitable chemotherapy strategy. We found that high-risk patients were more sensitive to Cisplatin, Docetaxel, and Elesclomol, while low-risk patients were more sensitive to Bicalutamide. Since Cisplatin, Docetaxel, and Bicalutamide are the three commonly used chemotherapy drugs for PCa in clinical practice, CRRS may help to select the appropriate chemotherapeutic agents. Elesclomol is a copper ionophore that can induce cuproptosis in cells (23), to which high-risk patients are more sensitive, further confirming the previously mentioned inhibitory state of cuproptosis in these patients. In the future, Elesclomol may be used to treat PCa under the premise of a reliable predictive biomarker.

In addition, we predicted six potential compounds, including purvalanol-A, aminopurvalanol-A, JAK3-inhibitor-VI, PHA-793887, Floxuridine, and Teniposide, for the treatment of PCa using the cMap Database. PHA-793887 significantly inhibited the growth of abiraterone-resistant PCa cell lines and patient-derived xenograft-derived PCa models (73). Floxuridine variants have potential therapeutic value in p53-mutated and hormone-dependent PCa (74). Purvalanol A can enhance the cytotoxic effect of taxol on non-small cell lung cancer cells *in vitro* through Op18/stathmin (75). Previous studies have shown that JAK3-inhibitor-VI is a promising candidate for treating acute myeloid leukemia (76). Teniposide has good efficacy in breast cancer (77). Aminopurvalanol-a has not been reported. In subsequent studies, we will explore the effects of these drugs on PCa treatment.

Finally, in PCa cell lines, the expression of these model genes was validated. All model genes were stably expressed in several PCa cell lines, except MYBPC1, which was detected only in PC3. MYBPC1 encodes a member of the myosin-binding protein C family, which may be expressed primarily in non-tumor cells in the TME. In addition, B4GALNT4 and FAM83D were significantly downregulated after induction of cuproptosis in most PCa cell lines, suggesting that these two genes are closely associated with cuproptosis activity in PCa cells. Studies have shown that FAM83D is strongly associated with cancer development, proliferation, invasion, and metastasis (78, 79). Beta-1,4-N-acetylgalactosaminyltransferase 4 (B4GALNT4), as an N-acetylgalactosamine transferase, is involved in the post-translational regulation of genes through protein glycosylation modifications (80). B4GALNT4 is upregulated in various cancers, and its expression can enhance the malignant potential of cancers (81). Considering that B4GALNT4 contributed the most to poor prognosis, we further investigated the model gene B4GALNT4. After the knockdown of B4GALNT4, the protein level of anti-cuproptosis CDKN2A was significantly down-regulated (23), indicating that the knockdown of B4GALNT4 might promote cuproptosis in PCa. Therefore, the up-regulation in the protein level of copper exporters ATP7A was probably due to the increase of copper ions in the cells after the enhancement of cuproptosis activity caused by the knockdown of B4GALNT4, and the compensatory up-regulation of ATP7A occurred in the cells to maintain the homeostasis of copper ions. Remarkably, the mRNA levels of the above CRGs were not significantly changed after the knockdown of B4GALNT4, suggesting that B4GALNT4 may regulate CRGs through post-transcriptional protein modifications. Meanwhile, transcriptomics analysis suggested that the knockdown of B4GALNT4 inhibited several classical pro-cancer pathways, including PI3K−Akt signaling and Wnt signaling pathways, indicating a pro-carcinogenic role of B4GALNT4 in PCa.

There are some limitations to our study. First of all, the signature was only validated with retrospective data. In the future, more prospective studies are required to verify its clinical value. Secondly, this study just investigated the relationship between the signature and TME as well as immunotherapy, only suggesting a possible correlation between them. Therefore, a clinical trial with sufficient samples to assess the value of this signature in guiding immunotherapy is required in the future. Thirdly, the value of the model for a personalized selection of chemotherapy drugs requires to be validated in later clinical trials, and the therapeutic effect of the screened potential small molecule compounds also needs to be further investigated. Lastly, further experiments *in vivo* and *in vitro* are required to investigate the role of the five model genes in PCa cuproptosis and tumorigenesis.

## 5 Conclusion

In conclusion, this study distinguished molecular subtypes based on CRGs in PCa and constructed a robust prognostic signature. The cuproptosis-related molecular subtypes and the prognostic signature could be used to predict the prognosis of PCa. Moreover, this signature may help to identify PCa patients who benefit more from anticancer immunotherapy and guide the choice of chemotherapy or targeted agents for patients with advanced PCa. In addition, we validated and explored the expression and regulation of model genes at the cellular level, respectively. Furthermore, the role of B4GALNT4 in cuproptosis and tumorigenesis in PCa was further explored through proteomics and transcriptomics analysis. In summary, our systematic study of CRGs will help to understand their role and value in PCa, and the signature can provide a reference for the clinical judgment of prognosis and selection of treatment options. Furthermore, we identified a potential cuproptosis-related oncogene in PCa, which could be a potential target to treat PCa in combination with cuproptosis.

## Data availability statement

The original contributions presented in the study are included in the article/**Supplementary Material**. Further inquiries can be directed to the corresponding authors.

## Author contributions

Conceptualization, JZ. Data curation, SJ. Funding acquisition, XG. Investigation, DG. Methodology, SJ. Project administration, XG. Resources, MQ. Software, WZ. Supervision, XS, MQ, CY and XG. Validation, DG. Writing – original draft, JZ. Writing – review & editing, YW. All authors contributed to the article and approved the submitted version.

## Glossary

PCa: Prostate cancer
RCD: Regulated cell death
CRGs: cuproptosis-related genes
TCGA: The Cancer Genome Atlas
DEGs: Differentially expressed genes
TME: The tumor microenvironment
ssGSEA: Single-sample gene set enrichment analysis
PFS: progression-free survival
ADT: Androgen deprivation therapy
CRPC: Castration-resistant PCa
TCA: tricarboxylic acid
UCSC: University of California, Santa Cruz
DFKZ: The German Cancer Research Center, Deutsches Krebsforschungszentrum
MSKCC: The Memorial Sloan Kettering Cancer Center
CPGEA: Chinese Prostate Cancer Genome and Epigenome Atlas
GEO: Gene Expression Omnibus
TMB: Tumor mutation burden
SCNA: somatic copy number alterations
K-M: Kaplan-Meier
GSVA: Gene Set Variation Analysis
GSEA: Gene Set Enrichment Analysis
MHC: Major histocompatibility complex
TIDE: Tumor Immune Dysfunction and Exclusion
LASSO: Least absolute shrinkage and selection operator
CRRS: Cuproptosis-related risk score
AUC: Area under the curve
ROC: Receiver operating characteristic
GDSC: Genomics of Drug Sensitivity in Cancer
IC50: The Half Maximal Inhibitory concentration
cMap: Connectivity Map
ANOVA: Analysis of variance
AIC: Akaike information criterion.
