# Supplementary figures and images for: Identification of novel molecular subtypes and a signature to predict prognosis and therapeutic response based on cuproptosis-related genes in prostate cancer
Source: Front Oncol. 2023 May 2;13:1162653. doi: 10.3389/fonc.2023.1162653 (PMC10185853; doi:10.3389/fonc.2023.1162653)

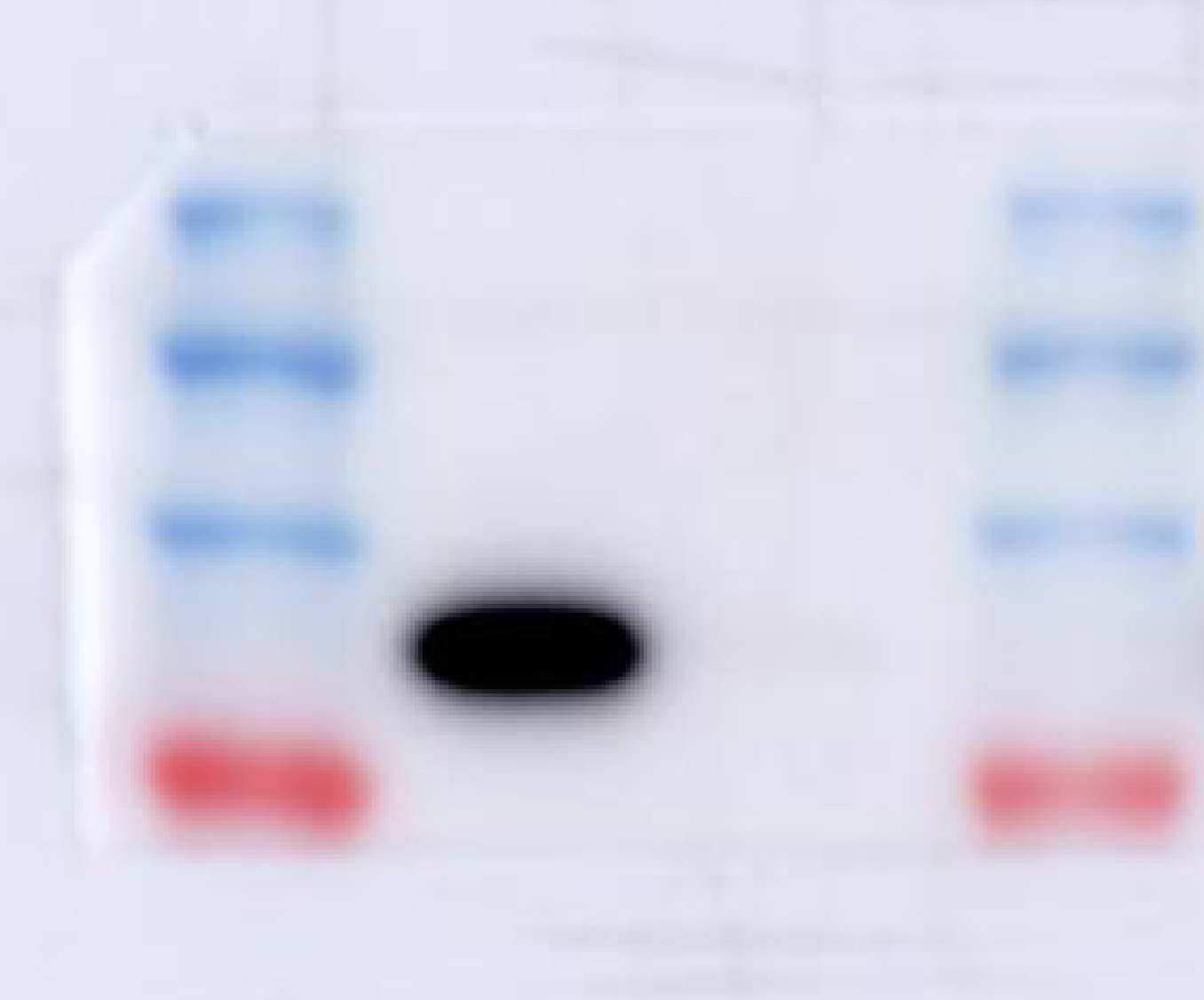

Supplement: Supplementary file 1 [file DataSheet_1.zip › Raw Data/03.Western Blot/replicate1/WB_B4GALNT4_1.jpg]

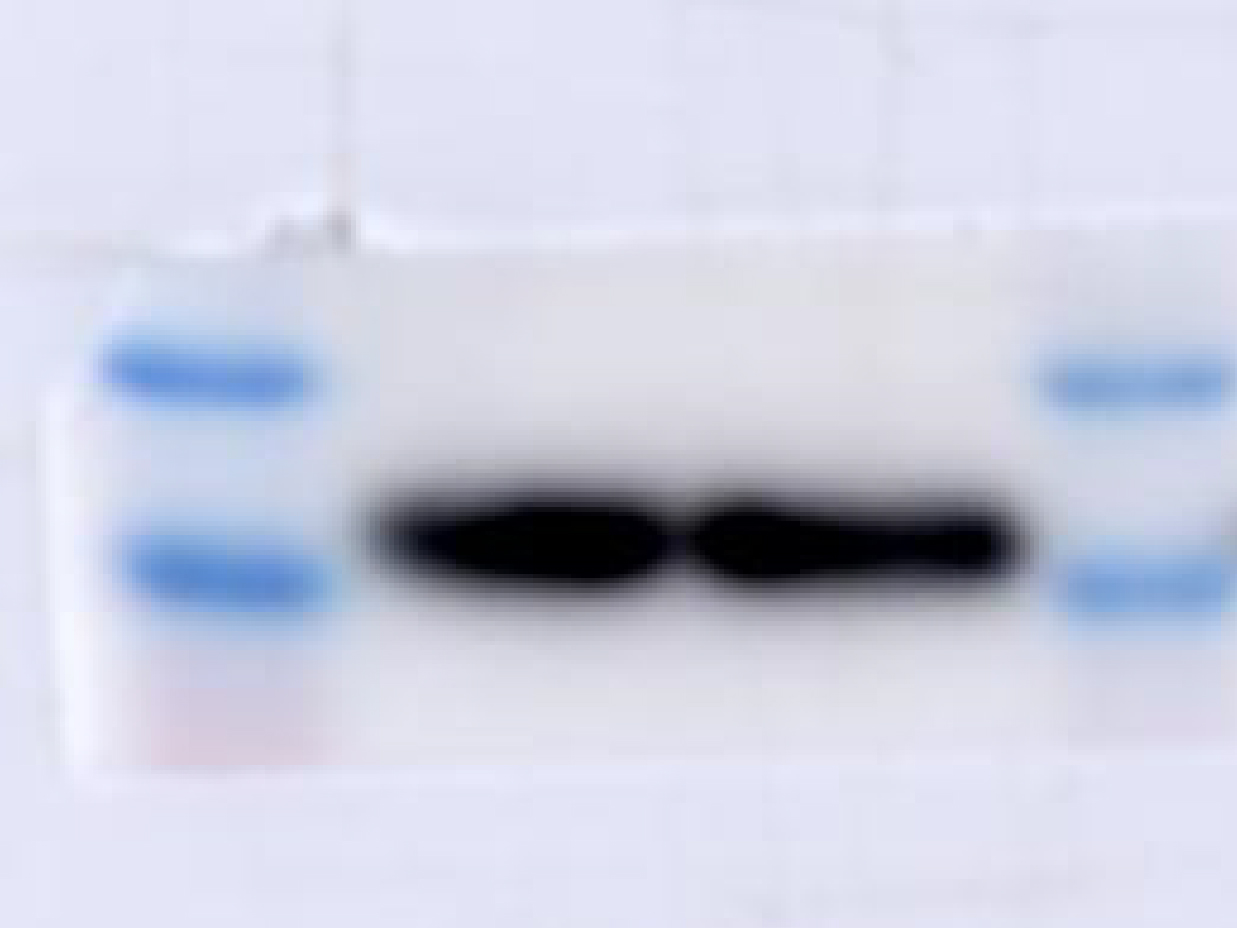

Supplement: Supplementary file 1 [file DataSheet_1.zip › Raw Data/03.Western Blot/replicate1/WB_GAPDH_1.jpg]

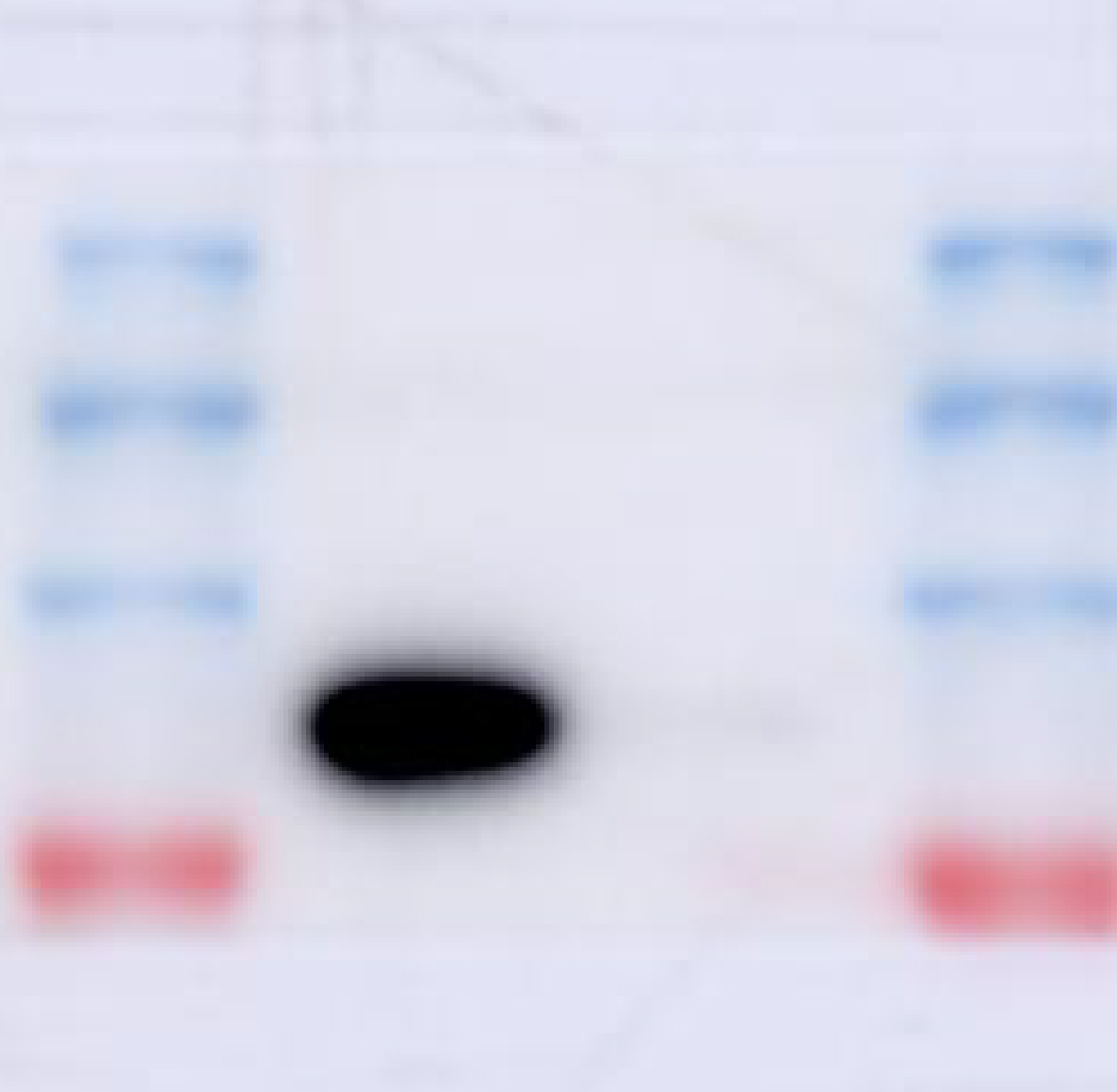

Supplement: Supplementary file 1 [file DataSheet_1.zip › Raw Data/03.Western Blot/replicate2/WB_B4GALNT4_2.jpg]

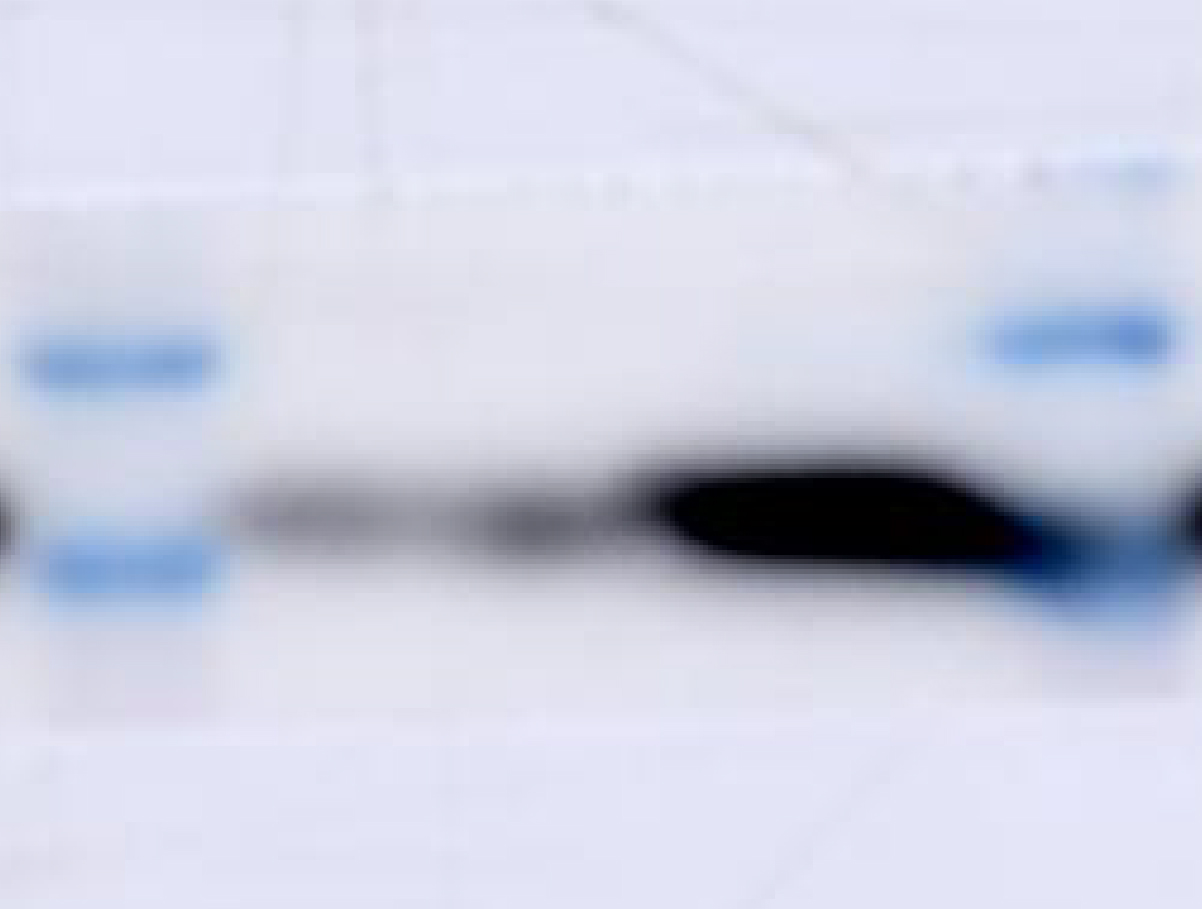

Supplement: Supplementary file 1 [file DataSheet_1.zip › Raw Data/03.Western Blot/replicate2/WB_GAPDH_12.jpg]

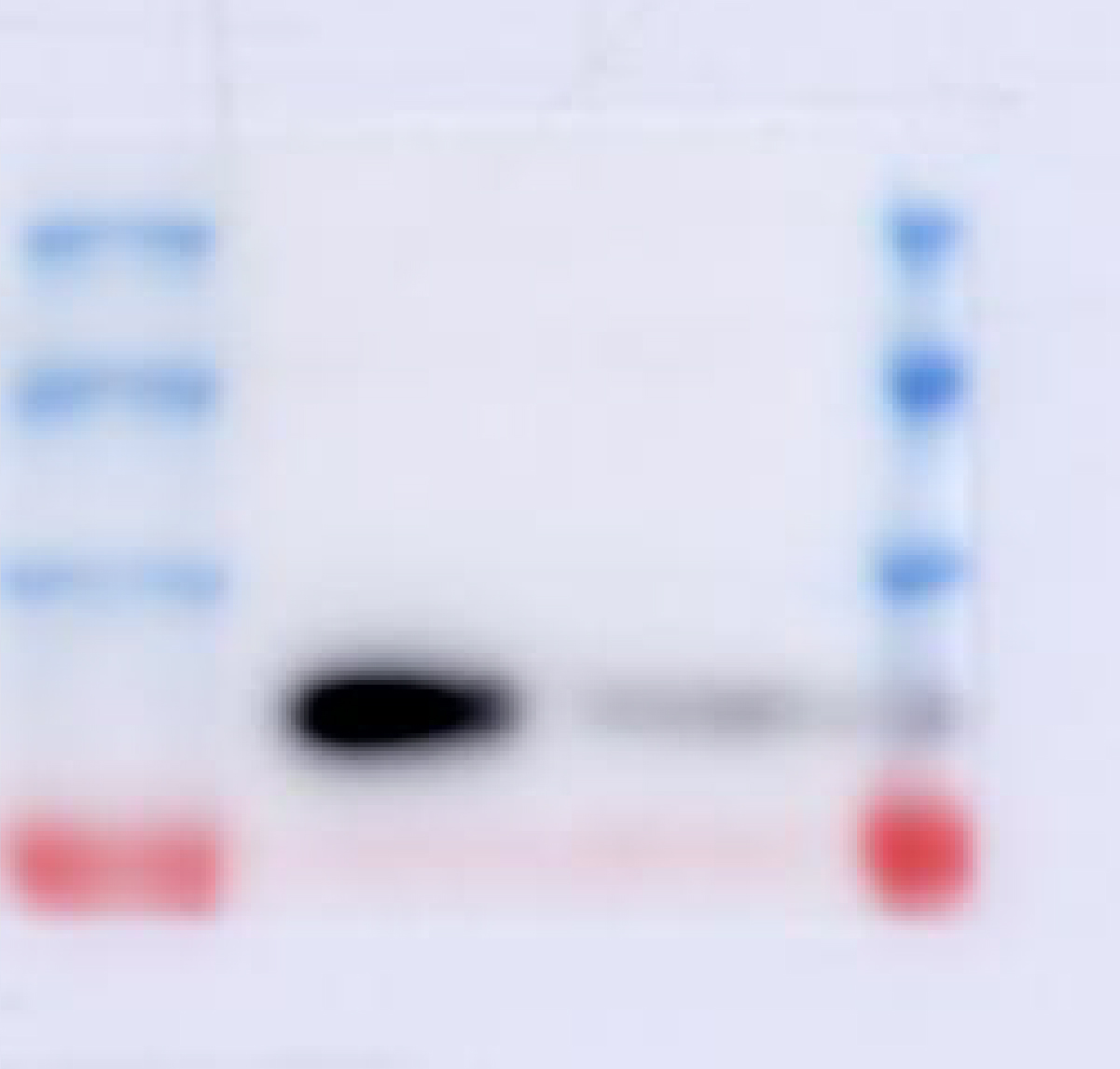

Supplement: Supplementary file 1 [file DataSheet_1.zip › Raw Data/03.Western Blot/replicate3/WB_B4GALNT4_3.jpg]

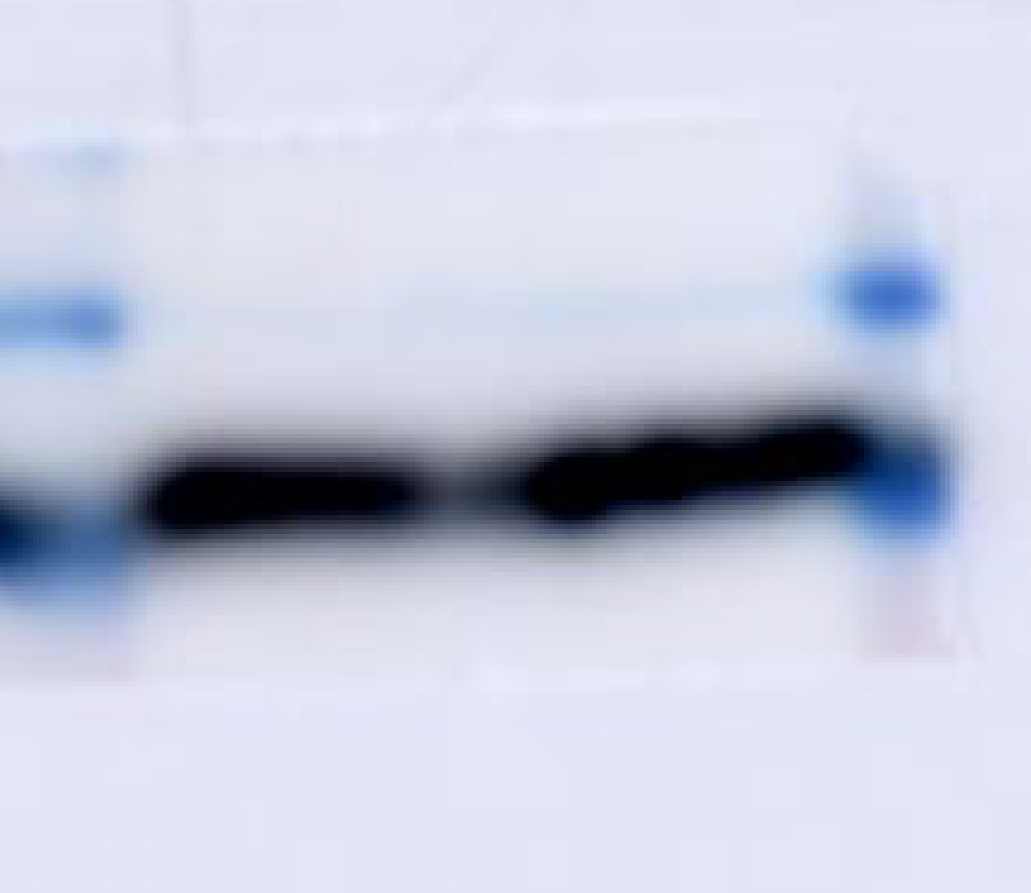

Supplement: Supplementary file 1 [file DataSheet_1.zip › Raw Data/03.Western Blot/replicate3/WB_GAPDH_3.jpg]

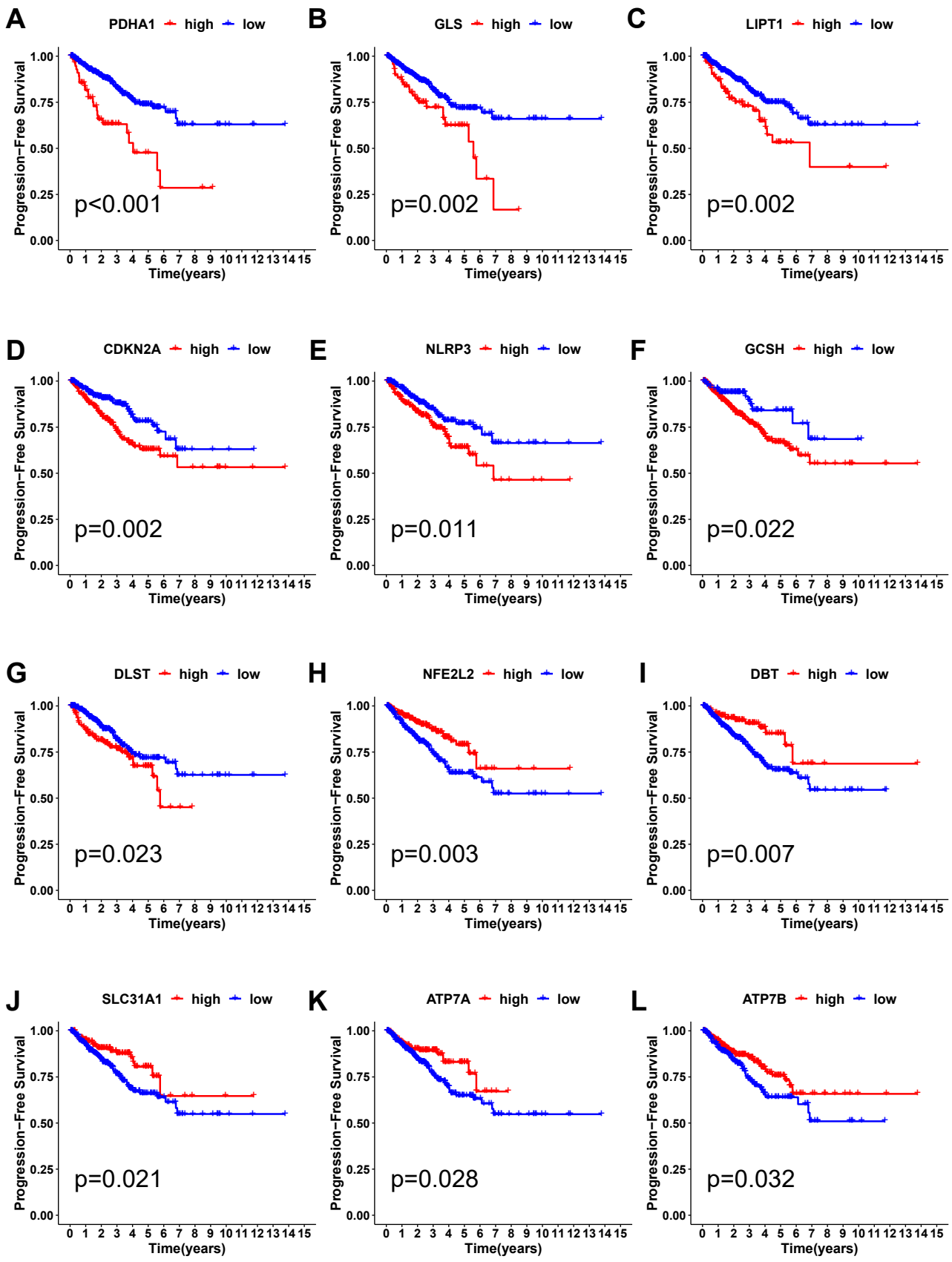

Supplement: Supplementary file 2 [file DataSheet_2.zip › supplementary figures&tables/Figure S1.pdf]

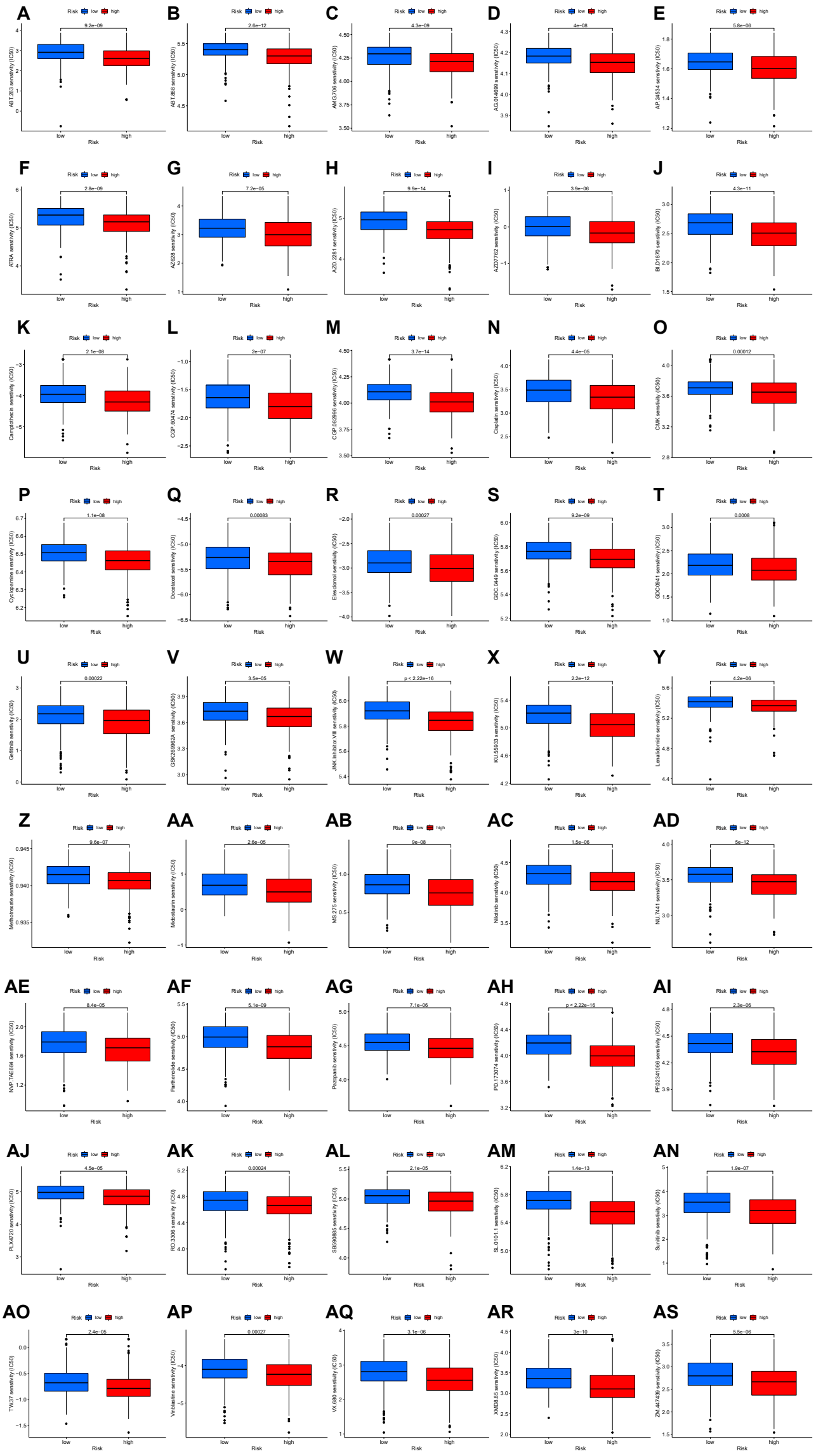

Supplement: Supplementary file 2 [file DataSheet_2.zip › supplementary figures&tables/Figure S4.pdf]

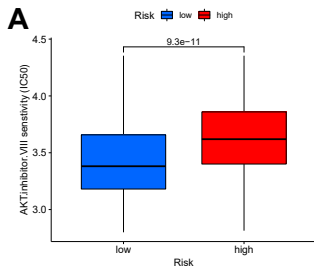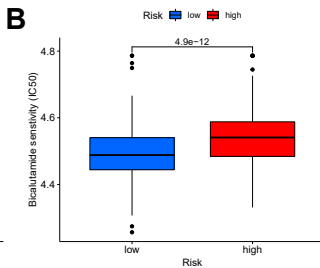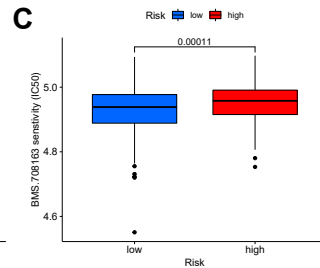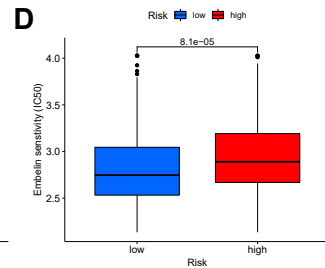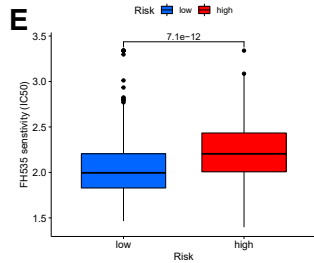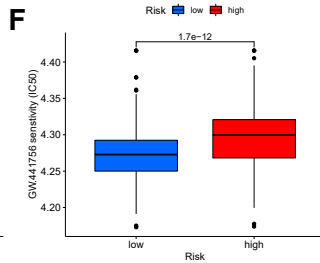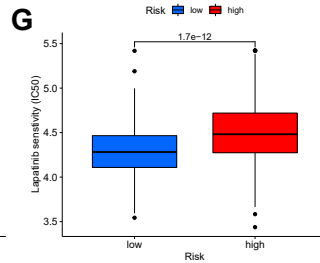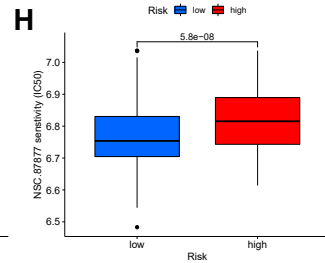

Supplement: Supplementary file 2 [file DataSheet_2.zip › supplementary figures&tables/Figure S5.pdf]
