# Supplementary material for: Identification of novel molecular subtypes and a signature to predict prognosis and therapeutic response based on cuproptosis-related genes in prostate cancer
Source: Front Oncol. 2023 May 2;13:1162653. doi: 10.3389/fonc.2023.1162653 (PMC10185853; doi:10.3389/fonc.2023.1162653)
Supplement: Supplementary file 2 [file DataSheet_2.zip › supplementary figures&tables/legends.docx]

**Figure S1.** (A-L) The Kaplan–Meier survival curve of PDHA1, GLS, LIPT1, CDKN2A, NLRP3, GCSH, DLST, NFE2L2, DBT, SLC31A1, ATP7A, and ATP7B.

**Figure S2.** (A) The genomic position and SCNA of CRGs, red means the SCNA frequency of gain is greater than that of loss, and blue means the SCNA frequency of loss is greater than that of gain. (B) The legend of the consensus matrix. (C-J) Consensus clustering matrix when k = 2-9. (K) Consensus clustering CDF with k valued 2 to 9. (L) Relative change in area under CDF curve for k = 2. (M) item tracking plot. (N) Box plot of the results of GSVA.

**Figure S3.** The heatmap (A) and boxplot (B)of the expression of immune checkpoint molecules between the two clusters. The heatmap (C) and boxplot (D) of the expression of genes that inhibit the cancer-immunity cycle between the two clusters. (E) The heatmap of the differentially expressed genes between the two clusters (Adjusted p<0.05 and | log2FC| > 0.585). (F) The comparison of risk score between cuproptosis subtypes.

**Figure S4.** (A-AS) Forty-five chemotherapy drugs with lower IC50 values in the high-risk group.

**Figure S5.** (A-H) Eight chemotherapy drugs with lower IC50 values in the low-risk group.

**Table S1.** The basic clinical characteristics of the nine cohorts of PCa.

**Table S2.** Gene sets for immune cells or immune fractions.

**Table S3.** The primers and oligonucleotides used in this study.

**Table S4.** Distribution of clinical features in training and test sets of TCGA cohort. **Table S5.** The differences in the chemotherapy response of common chemotherapy drugs between the two risk groups.
