# Supplementary material for: Identification of novel molecular subtypes and a signature to predict prognosis and therapeutic response based on cuproptosis-related genes in prostate cancer
Source: Front Oncol. 2023 May 2;13:1162653. doi: 10.3389/fonc.2023.1162653 (PMC10185853; doi:10.3389/fonc.2023.1162653)
Supplement: Supplementary file 2 [file DataSheet_2.zip › supplementary figures&tables/Table S1.docx]

**Table S1** | The basic clinical features of the nine cohorts of PCa.

| **Features** |  | | | | **N (%)** | | | | | |
| --- | --- | --- | --- | --- | --- | --- | --- | --- | --- | --- |
|  | **TCGA**  **(n=497)** | **DKFZ (n=81)** | **MSKCC (n=140)** | **CPGEA (n=125)** | | **GSE46602 (n=36)** | **GSE70768 (n=111)** | **GSE70769 (n=92)** | **GSE70770 (n=203)** | **GSE54460 (n=91)** |
| **Age, years** |  |  |  |  | |  |  |  |  |  |
| <=60 | 223(44.87) | 81(100.00) | 0(0.00) | 12(9.60) | | 15(41.67) | 48(43.24) | 0(0.00) | 48(23.65) | 40(43.96) |
| >60 | 274(55.13) | 0(0.00) | 0(0.00) | 113(90.40) | | 21(58.33) | 63(56.76) | 0(0.00) | 63(31.03) | 51(56.04) |
| unknown | 0(0.00) | 0(0.00) | 140(100.00) | 0(0.00) | | 0(0.00) | 0(0.00) | 92(100.00) | 92(45.32) | 0(0.00) |
| **GS** |  |  |  |  | |  |  |  |  |  |
| <=6 | 45(9.05) | 11(13.58) | 0(0.00) | 10(8.00) | | 17(47.22) | 17(15.32) | 20(21.74) | 37(18.23) | 11(12.09) |
| 7 | 247(49.70) | 60(74.07) | 0(0.00) | 59(47.20) | | 15(41.67) | 85(76.58) | 55(59.78) | 140(68.97) | 69(75.82) |
| 8 | 64(12.88) | 1(1.23) | 0(0.00) | 20(16.00) | | 3(8.33) | 8(7.20) | 5(5.43) | 13(6.40) | 8(8.79) |
| 9 | 137(27.57) | 8(9.88) | 0(0.00) | 35(28.00) | | 1(2.78) | 1(0.90) | 9(9.78) | 10(4.93) | 3(3.30) |
| 10 | 4(0.80) | 1(1.23) | 0(0.00) | 0(0.00) | | 0(0.00) | 0(0.00) | 1(1.09) | 1(0.49) | 0(0.00) |
| unknown | 0(0.00) | 0(0.00) | 140(100.00) | 1(0.80) | | 0(0.00) | 0(0.00) | 2(2.17) | 2(0.99) | 0(0.00) |
| **pT stage** |  |  |  |  | |  |  |  |  |  |
| <=T2 | 187(37.63) | 55(67.90) | 86(61.43) | 64(51.20) | | 19(52.78) | 34(30.63) | 48(52.17) | 82(40.39) | 75(82.42) |
| T3 | 293(58.95) | 23(28.40) | 47(33.57) | 57(45.60) | | 17(47.22) | 76(68.47) | 42(45.65) | 118(58.13) | 14(15.38) |
| T4 | 10(2.01) | 3(3.70) | 7(5.00) | 4(3.20) | | 0(0.00) | 1(0.90) | 0(0.00) | 1(0.49) | 1(1.10) |
| unknown | 7(1.41) | 0(0.00) | 0(0.00) | 0(0.00) | | 0(0.00) | 0(0.00) | 2(2.17) | 2(0.99) | 1(1.10) |
| **pN stage** |  |  |  |  | |  |  |  |  |  |
| N0 | 345(69.42) | 0(0.00) | 0(0.00) | 89(71.20) | | 0(0.00) | 82(73.87) | 18(19.57) | 100(49.26) | 35(38.46) |
| N1 | 79(15.9) | 0(0.00) | 0(0.00) | 15(12.00) | | 0(0.00) | 8(7.21) | 0(0.00) | 8(3.94) | 0(0.00) |
| unknown | 73(14.69) | 81(100.00) | 140(100.00) | 21(16.80) | | 36(100.00) | 21(18.92) | 74(80.43) | 95(46.80) | 56(61.54) |
| **M stage** |  |  |  |  | |  |  |  |  |  |
| M0 | 455(91.55) | 0(0.00) | 0(0.00) | 111(88.80) | | 0(0.00) | 20(18.02) | 26(28.26) | 32(15.76) | 3(3.30) |
| M1 | 3(0.60) | 0(0.00) | 0(0.00) | 10(8.00) | | 0(0.00) | 1(0.90) | 4(4.35) | 5(2.46) | 0(0.00) |
| unknown | 39(7.85) | 81(100.00) | 140(100.00) | 4(3.20) | | 36(100.00) | 90(81.08) | 62(67.39) | 166(81.77) | 88(96.70) |

*PCa, Prostate cancer; TNM, tumor node metastasis; GS,* *Gleason score; p, pathology.*
