# Supplementary material for: Identification of novel molecular subtypes and a signature to predict prognosis and therapeutic response based on cuproptosis-related genes in prostate cancer
Source: Front Oncol. 2023 May 2;13:1162653. doi: 10.3389/fonc.2023.1162653 (PMC10185853; doi:10.3389/fonc.2023.1162653)
Supplement: Supplementary file 2 [file DataSheet_2.zip › supplementary figures&tables/Table S3.docx]

| **Primer Name** | **Forward** | **Reverse** |
| --- | --- | --- |
| B4GALNT4 | 5'-ACTGGGAGCTCCTGGACA-3' | 5'-TGGTGATAGAAATTCCGCAGT-3' |
| FAM83D | 5'-GGCAACAGGCTCCTACAGTTTTA-3' | 5'-GACAGGAGTTTGGGGCTGAT-3' |
| COL1A1 | 5'-CCCCGAGGCTCTGAAGGT-3' | 5'-GCAATACCAGGAGCACCATTG-3' |
| CHRM3 | 5'-AGCAGCAGTGACAGTTGGAAC -3' | 5'-CTTGAGCACGATGGAGTAGATGG-3' |
| MYBPC1 | 5'-GCATGCTCAAGCGACTCAAG-3' | 5'-CTGCCAGCTCCACAACAAAC-3' |
| Homo β-actin | 5'-CGCGAGAAGATGCCCAGATC-3' | 5'-TCACCGGAGTCCATCACGA-3' |
| shB4GALNT4 | 5'-CCGGGCAATTTGTGTACCTGTCCTTCTCGAGAAGGACAGGTACACAAATTGCTTTTTG-3' | 5'-AATTCAAAAAGCAATTTGTGTACCTGTCCTTCTCGAGAAGGACAGGTACACAAATTGC-3' |
| shControl | 5'-CCGGCAACAAGATGAAGAGCACCAACTCGAGTTGGTGCTCTTCATCTTGTTGTTTTTG-3' | 5'-AATTCAAAAACAACAAGATGAAGAGCACCAACTCGAGTTGGTGCTCTTCATCTTGTTG-3' |

**Table S3**. The primers and oligonucleotides used in this study.

shRNA, short hairpin RNA.
