# Supplementary material for: Identification of novel molecular subtypes and a signature to predict prognosis and therapeutic response based on cuproptosis-related genes in prostate cancer
Source: Front Oncol. 2023 May 2;13:1162653. doi: 10.3389/fonc.2023.1162653 (PMC10185853; doi:10.3389/fonc.2023.1162653)
Supplement: Supplementary file 2 [file DataSheet_2.zip › supplementary figures&tables/Table S4.docx]

**TABLE S4 |** Distribution of clinical features in training and test sets of TCGA cohort.

| **Characteristics** | **N (%)**  **Entire dataset**  **(n=497)** | **N (%)** | | ***P*** |
| --- | --- | --- | --- | --- |
|  |  | **Training group**  **(n=249)** | **Test group**  **(n=248)** |  |
| **Age,years** |  |  |  | 0.825 |
| <=60 | 223(44.87) | 110(44.18) | 113(45.56) |  |
| >60 | 274(55.13) | 139(55.82) | 135(54.44) |  |
| **Gleason score** |  |  |  | 0.673 |
| 6 | 45(9.05) | 19(7.63) | 26(10.48) |  |
| 7 | 247(49.70) | 126(50.60) | 121(48.79) |  |
| 8 | 64(12.88) | 29(11.65) | 35(14.11) |  |
| 9 | 137(27.57) | 73(29.32) | 64(25.81) |  |
| 10 | 4(0.80) | 2(0.80) | 2(0.81) |  |
| **pT stage** |  |  |  | 0.859 |
| T2 | 187(37.63) | 91(36.55) | 96(38.71) |  |
| T3 | 293(58.95) | 149(59.84) | 144(58.06) |  |
| T4 | 10(2.01) | 6(2.41) | 4(1.61) |  |
| unknown | 7(1.41) | 3(1.20) | 4(1.61) |  |
| **pN stage** |  |  |  | 0.975 |
| N0 | 345(69.42) | 174(69.88) | 171(68.95) |  |
| N1 | 79(15.90) | 39(15.66) | 40(16.13) |  |
| unknown | 73(14.69) | 36(14.46) | 37(14.92) |  |
| **M stage** |  |  |  | 0.598 |
| M0 | 455(91.55) | 225(90.36) | 230(92.74) |  |
| M1 | 3(0.60) | 2(0.80) | 1(0.40) |  |
| unknown | 39(7.85) | 22(8.84) | 17(6.85) |  |

*TCGA, the Cancer Genome Atlas; TNM, tumor node metastasis; p, pathology.*
