# Supplementary material for: Identification of novel molecular subtypes and a signature to predict prognosis and therapeutic response based on cuproptosis-related genes in prostate cancer
Source: Front Oncol. 2023 May 2;13:1162653. doi: 10.3389/fonc.2023.1162653 (PMC10185853; doi:10.3389/fonc.2023.1162653)
Supplement: Supplementary file 2 [file DataSheet_2.zip › supplementary figures&tables/Table S5.docx]

**Table S5**. The differences in the chemotherapy response of common chemotherapy drugs between the two risk groups.

| **Drug** | **Group with lower IC50** | ***P* value** | **Drug** | **Group with lower IC50** | ***P* value** | **Drug** | **Group with lower IC50** | ***P* value** |
| --- | --- | --- | --- | --- | --- | --- | --- | --- |
| ABT.263 | high | 9.2e−09 | GDC.0449 | high | 9.2e−09 | RO.3306 | high | 2.4e−04 |
| ABT.888 | high | 2.6e−12 | GDC0941 | high | 8.0e−04 | SB590885 | high | 2.1e−05 |
| AG.014699 | high | 4.0e−08 | Gefitinib | high | 2.2e−04 | SL.0101.1 | high | 1.4e−13 |
| AMG.706 | high | 4.3e−09 | GSK269962A | high | 3.5e−05 | Sunitinib | high | 1.9e−07 |
| AP.24534 | high | 5.8e−06 | JNK.Inhibitor.VIII | high | <2.2e−16 | TW.37 | high | 2.4e−05 |
| ATRA | high | 2.8e−09 | KU.55933 | high | 2.2e−12 | Vinblastine | high | 2.7e−04 |
| AZ628 | high | 7.2e−05 | Lenalidomide | high | 4.2e−06 | VX.680 | high | 3.1e−06 |
| AZD.2281 | high | 9.9e−14 | Methotrexate | high | 9.6e−07 | XMD8.85 | high | 3.0e−10 |
| AZD7762 | high | 3.9e−06 | Midostaurin | high | 2.6e−05 | ZM.447439 | high | 5.5e−06 |
| BI.D1870 | high | 4.3e−11 | MS.275 | high | 9.0e−08 | AKT.inhibitor.VIII | low | 9.3e−11 |
| Camptothecin | high | 2.1e−08 | Nilotinib | high | 1.5e−06 | Bicalutamide | low | 4.9e−12 |
| CGP.082996 | high | 3.7e−14 | NU.7441 | high | 5.0e−12 | BMS.708163 | low | 1.1e−04 |
| CGP.60474 | high | 2.0e−07 | NVP.TAE684 | high | 8.4e−05 | Embelin | low | 8.1e−05 |
| Cisplatin | high | 4.4e−05 | Parthenolide | high | 5.1e−09 | FH535 | low | 7.1e−12 |
| CMK | high | 1.2e−04 | Pazopanib | high | 7.1e−06 | GW.441756 | low | 1.7e−12 |
| Cyclopamine | high | 1.1e−08 | PD.173074 | high | <2.2e−16 | Lapatinib | low | 1.7e−12 |
| Docetaxel | high | 8.3e−04 | PF.02341066 | high | 2.3e−06 | NSC.87877 | low | 5.8e−08 |
| Elesclomol | high | 2.7e−04 | PLX4720 | high | 4.5e−05 |  |  |  |

*IC50, The Half Maximal Inhibitory concentration.*
