# Supplementary material for: Identification of novel molecular subtypes and a signature to predict prognosis and therapeutic response based on cuproptosis-related genes in prostate cancer
Source: Front Oncol. 2023 May 2;13:1162653. doi: 10.3389/fonc.2023.1162653 (PMC10185853; doi:10.3389/fonc.2023.1162653)
Supplement: Supplementary file 2 [file Figure S2.pdf › supplementary figures&tables/Figure S2.pdf]

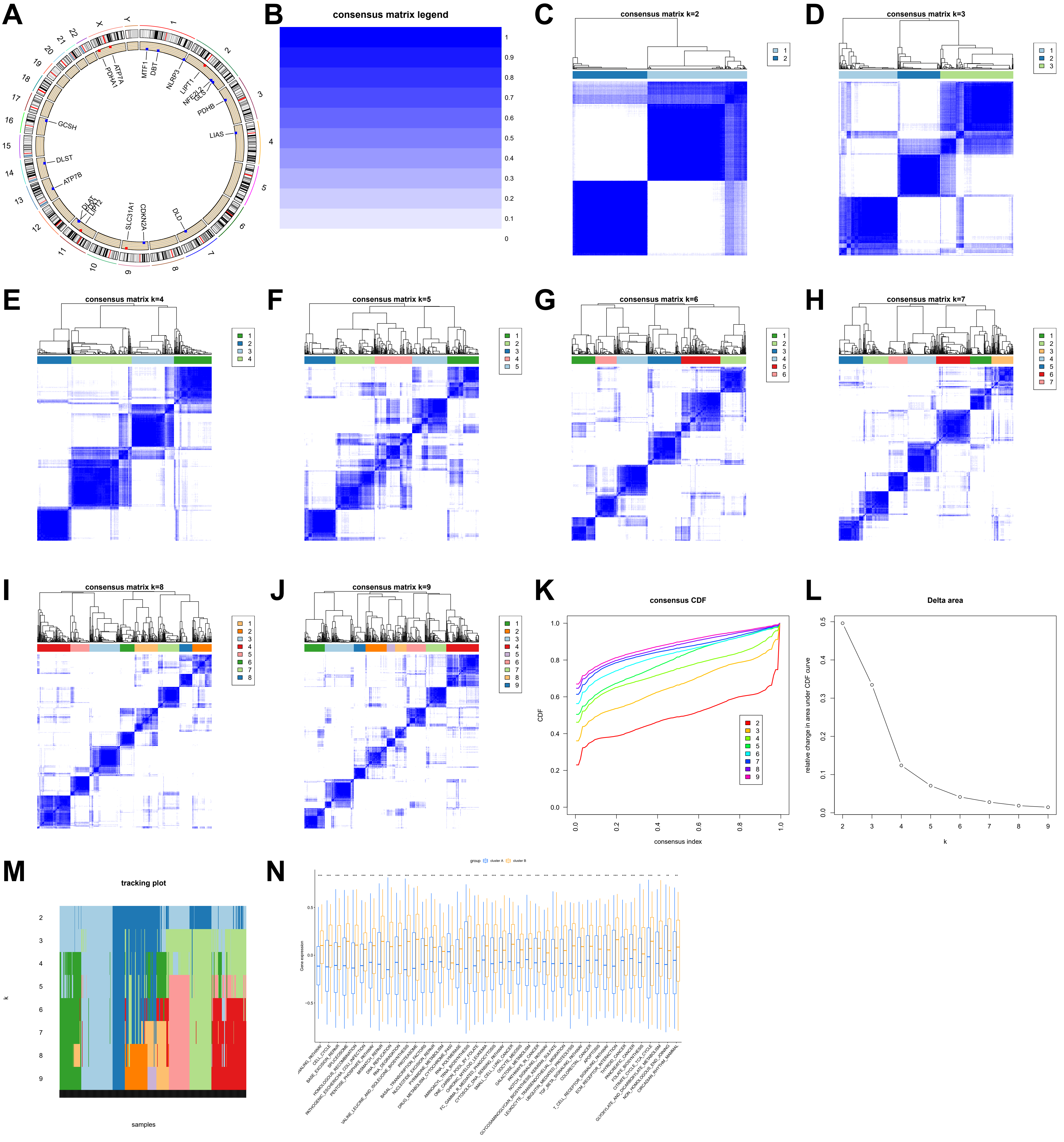
A
MTF1
DBT
NLRP3
LIPT1
NFE2L2
GLS
PDHB
LIAS
DLD
CDKN2A
SLC31A1
DLAT
LIPA2
ATP7B
DLST
GCSH
PDHA1
ATP7A
B
consensus matrix legend
C
consensus matrix k=2
D
consensus matrix k=3
E
consensus matrix k=4
F
consensus matrix k=5
G
consensus matrix k=6
H
consensus matrix k=7
I
consensus matrix k=8
J
consensus matrix k=9
K
consensus CDF
CDF
consensus index
L
Delta area
relative change in area under CDF curve
k
M
tracking plot
k
samples
N
group
cluster A
cluster B
Gene expression
