# Supplementary material for: Identification of novel molecular subtypes and a signature to predict prognosis and therapeutic response based on cuproptosis-related genes in prostate cancer
Source: Front Oncol. 2023 May 2;13:1162653. doi: 10.3389/fonc.2023.1162653 (PMC10185853; doi:10.3389/fonc.2023.1162653)
Supplement: Supplementary file 2 [file Figure S3.pdf › supplementary figures&tables/Figure S3.pdf]

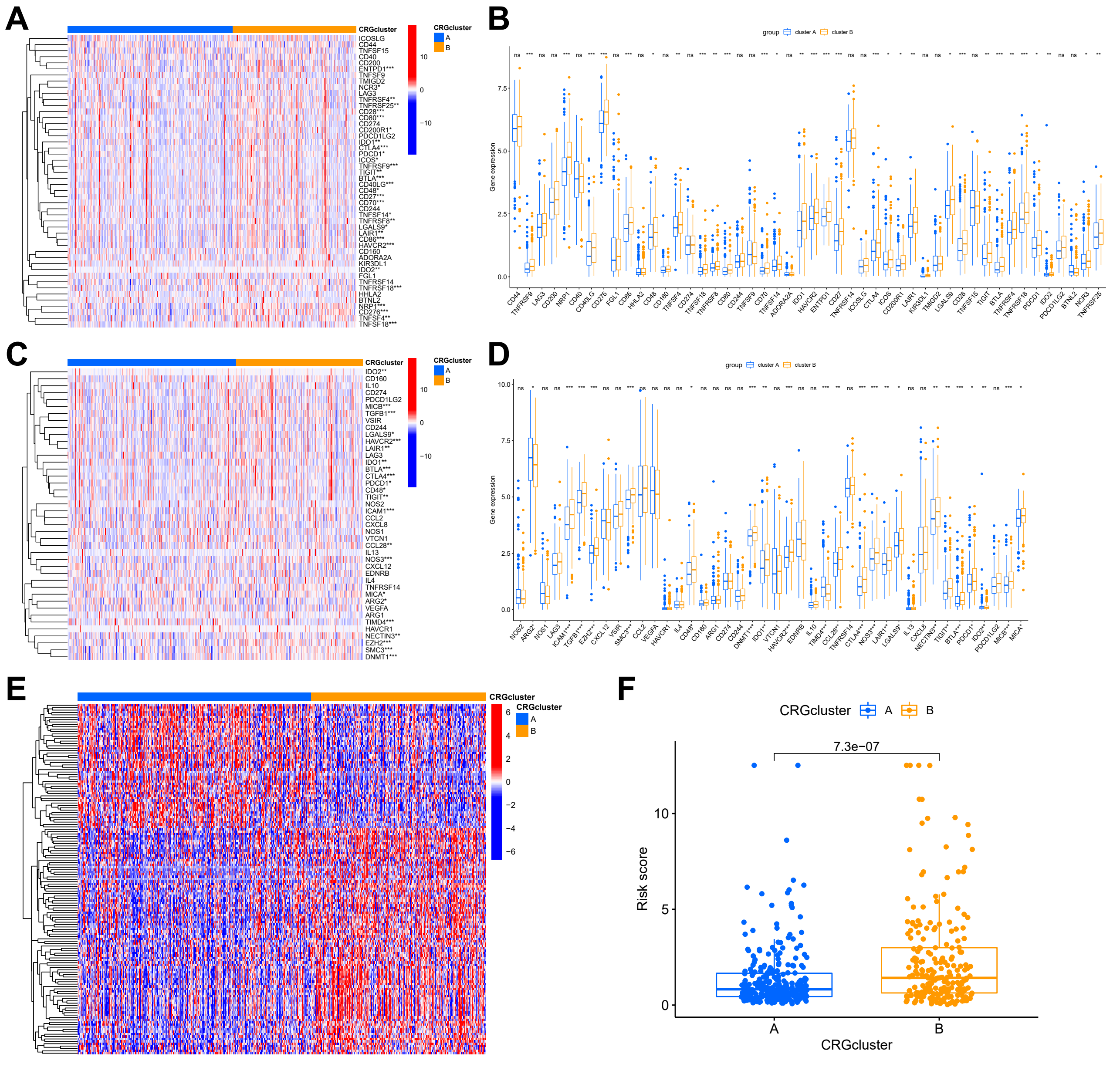

A
CRGcluster
A
B
10
0
−10
ICOSLG
CD44
TNFSF15
CD40
CD200
ENTPD1***
TNFSF9
TMIGD2
NCR3*
LAG3
TNFRSF4**
TNFRSF25**
CD28***
CD80***
CD274
CD200R1*
PDCD1LG2
IDO1**
CTLA4***
PDCD1*
ICOS*
TNFRSF9***
TIGIT**
BTLA***
CD40LG***
CD48*
CD27***
CD70***
CD244
TNFSF14*
TNFRSF8**
LGALS9*
LAIR1**
CD86***
HAVCR2***
CD160
ADORA2A
KIR3DL1
IDO2**
FGL1
TNFRSF14
TNFRSF18***
HHLA2
BTNL2
NRP1***
CD276***
TNFSF4**
TNFSF18***
B
group
cluster A
cluster B
Gene expression
0.0
2.5
5.0
7.5
CD44
TNFRSF9
LAG3
CD200
NRP1
CD40
CD40LG
CD276
FGL1
CD86
HHLA2
CD48
CD160
TNFSF4
CD274
TNFSF18
TNFRSF8
CD80
CD244
TNFSF9
CD70
TNFSF14
ADORA2A
IDO1
HAVCR2
ENTPD1
CD27
TNFRSF14
ICOSLG
CTLA4
ICOS
CD200R1
LAIR1
KIR3DL1
TMIGD2
LGALS9
CD28
TNFSF15
TIGIT
BTLA
TNFRSF4
TNFRSF18
PDCD1
IDO2
PDCD1LG2
BTNL2
NCR3
TNFRSF25
C
CRGcluster
A
B
10
0
−10
IDO2**
CD160
IL10
CD274
PDCD1LG2
MICB***
TGFB1***
VSIR
CD244
LGALS9*
HAVCR2***
LAIR1**
LAG3
IDO1**
BTLA***
CTLA4***
PDCD1*
CD48*
TIGIT**
NOS2
ICAM1***
CCL2
CXCL8
NOS1
VTCN1
CCL28**
IL13
NOS3***
CXCL12
EDNRB
IL4
TNFRSF14
MICA*
ARG2*
VEGFA
ARG1
TIMD4***
HAVCR1
NECTIN3**
EZH2***
SMC3***
DNMT1***
D
group
cluster A
cluster B
Gene expression
0.0
2.5
5.0
7.5
10.0
NOS2
ARG2*
NOS1
LAG3
ICAM1***
TGFB1***
EZH2***
CXCL12
VSIR
SMC3***
CCL2
VEGFA
HAVCR1
IL4
CD48*
CD160
ARG1
CD274
CD244
DNMT1***
IDO1**
VTCN1
HAVCR2***
EDNRB
IL10
TIMD4***
CCL28**
TNFRSF14
CTLA4***
NOS3***
LAIR1**
LGALS9*
IL13
CXCL8
NECTIN3**
TIGIT**
BTLA***
PDCD1*
IDO2**
PDCD1LG2
MICB***
MICA*
E
CRGcluster
A
B
6
4
2
0
−2
−4
−6
F
CRGcluster
A
B
7.3e−07
Risk score
0
5
10
CRGcluster
